# A scoping review to map public-facing websites for non-traumatic wrist disorders with quality evaluation

**DOI:** 10.1177/17589983241287082

**Published:** 2024-10-08

**Authors:** Thomas Mitchell, Michael Bircumshaw, Clare Cryan, Dawid Kotwica, Nick Hamilton, Ben Dean, Sionnadh McLean

**Affiliations:** 1 Health Research Institute, 111995 Sheffield Hallam University, Sheffield, UK; 2 Nuffield Department of Clinical Neurosciences, 159064 University of Oxford, Oxford, UK; 3 627148 Health Science, Charles Darwin University, Darwin, Australia

**Keywords:** Non-traumatic wrist disorder, wrist pain, patient-information websites, health information, wrist injury

## Abstract

**Introduction:**

Public-facing resources for non-traumatic wrist disorders (NTWD) exist, but care recipients and healthcare professionals alike are uncertain where to access the most useful resources and have raised concerns over the quality of information provided. Previous studies involving stakeholders highlight a need for quality evaluation of these resources. The aim of this study was to identify website resources accessible to UK-based online health seekers and explore their content through quality assessment.

**Methods:**

A scoping review of public-facing websites was conducted in accordance with Joanna Briggs Institute guidelines and PRISMA-ScR checklist. An *a-priori* search strategy was performed of publicly accessible websites using lay terms were entered into a simple Google search. The DISCERN tool was used to appraise the quality of health information with additional data charted to pre-determined criteria.

**Results:**

The 82 websites meeting inclusion criteria scored an average of 2/5 DISCERN. Nine funding categories existed with private service websites were the most common. 18 different diagnoses were found with twenty different management interventions were recommended.

**Conclusion:**

Considerable variation was found in the quality of websites providing information which people with NTWD are likely to access. Quality and trustworthiness of website information on NTWD are not the preserve of any sector or organisation and we identified potential for improvements across the board.

## Introduction

Reliable online information has been cited as a means to aid collaboration between recipients of care and practitioners through goal setting and the reinforcement of patient education messages.^1^ As models of healthcare provision move toward empowering individuals as active participants in their healthcare through the holistic ‘people-centred’ model recommended by the World Health Organisation^2^ or the ‘person-centred’ model of care recommended in NHS Long Term Plan,^3^ the importance of easily accessible and trusted information to aid an individual’s health choices are heightened.

Public-facing websites are an increasingly accessible resource with 94% of households found to have access to computer networks in the United Kingdom (UK) in 2021.^4^ It is common for individuals to use online resources for health information with 68% of people reporting researching symptoms online prior to attending medical appointments.^5^ This has significance as there is evidence to suggest online health-seeking behaviour can influence individuals offline health-related intentions, decision making and behaviours.^5,6^ Opportunities exist both for clinicians to guide recipients of care to credible online sources to assist in management, and for providers of healthcare services or products to influence the recipients of care’s decision making. However, it is known that low regulation of online health resources has allowed misleading or incorrect information to be published leading to ambiguity of message and encouraging potentially harmful behaviours.^7,8^

Uncertainty in identifying reliable and useful information online about wrist ganglion, tendinopathies, ulna-sided wrist pain, osteoarthritis and instabilities, grouped as non-traumatic wrist disorders (NTWD) has been identified by stakeholders.^9^ These conditions bear a considerable burden of personal and organisational cost whilst being resistant to clear pathological or anatomical explanations for their associated pain and disability.^10–15^ Non-specific wrist pain is common in the UK adult population with an estimated annual consultation rate of 58/10,000^16^ and thought to account for 10% of all musculoskeletal pain consultations in UK primary care.^17^ In specialist secondary care hand and wrist clinics in the UK, 13% of referrals were found to be for non-traumatic wrist pain.^18^

Difficulty for recipients of care and those charged with their management to find trustworthy online resources may impact attempts to promote holistic person-centred care. There is a clear need to identify and appraise available online information for musculoskeletal disorders generally, and NTWD specifically, to allow investigation into how website content aligns with best available evidence. Scoping review methodology has been used previously to examine the messaging of public-facing websites for diagnostic imaging for adults with low back pain, knee pain, and shoulder pain,^19^ however we were unable to identify any investigation into musculoskeletal wrist problems. Scoping review methodology has been chosen to investigate public facing websites for NTWD as it allows non-published sources to be appraised and the landscape of evidence for poorly understood areas to be observed and recorded.^20^ In describing written data held on public-facing websites, extracting data and assessing its quality, areas of unwarranted variation can be identified and recommendations for improvement in online materials can be made. The aim of this study was to identify website resources accessible to UK-based online health seekers and explore their content through quality assessment.

### Review objectives

• To identify public-facing websites displaying healthcare information for people with NTWD.
• To describe and summarise website written content using a data extraction tool.
• To perform a quality assessment of extracted data using the DISCERN^21^ tool.

## Methods

A protocol for the review was registered on the Open Science Framework on 10 March 2023 prior to conducting searches (https://osf.io/xc57r). The authorship has been amended from the initial proposal to reflect contributions to the project. Our intention was to perform reflexive thematic analysis. However, we judged the reflexive component of our analysis as insufficiently rigorous and reverted instead to basic thematic analysis.

### Study team composition

The review team was comprised of a PhD student and three MSc students at Sheffield Hallam University, academics, and subject area specialists.

### Scoping review framework

Scoping review methodology allows a systematic approach to map evidence into poorly understood areas^22^ and may draw on evidence from non-empirical research studies including grey literature sources. This study used the Joanna Briggs Institute (JBI) methodology^22^ for scoping reviews and the PRISMA-ScR checklist.^20^ As scoping reviews record the nature of evidence available and are not primary research, ethical approval was not sought for this study.

### Eligibility criteria

Websites were included if they were in the English language, created to provide information on any of the individual or grouped conditions identified about under the term NTWD and were freely accessible on Google search. There was no time cut-off for the creation of the webpages. Websites that had multiple text-based pages, links and PDF resources hosted within the website were included.

Exclusion criteria were: video sharing platforms (e.g. YouTube), social media links, audio links, websites not publicly accessible or behind paywalls, sponsored listings, clinician- or academic-facing websites and articles (e.g. scientific journals), resources for rheumatoid arthritis,^23^ carpal tunnel syndrome,^24–26^ complex regional pain syndrome,^27^ base of thumb osteoarthritis, and hand, thumb or finger injuries. Websites using ‘hand’ and ‘wrist’ interchangeably and those referring to multiple wrist conditions were included.

### Search strategy

General search engines were used in line with previous findings that these were used by majority of health seekers to obtain information about their condition.^28^ The Google Chrome web browser was used as it is the most commonly used in the UK (49.8% of internet users) and a Google search engine was chosen as it has a share of 83.5% of all UK web searches.^29^ An iterative process in the search strategy attempted to replicate the way a UK citizen with NTWD may look for information about their conditions. ‘Lay terms’ were extracted from recipients of care responses in the mixed stakeholder group conducted by Mitchell et al,^9^ entered into a Google search and suggested autocomplete terms were recorded as a means of reflecting how people may phrase their online searches (Supplementary section 1).

To eliminate the effect of online cookies, the search was completed in incognito mode on Google Chrome. The six search terms were divided up between the three researchers and searches were performed on 16 March 2023 with the first 50 returned websites recorded. To ensure the first 50 hits remained constant throughout the review process, the website domain and title were recorded in a spreadsheet (Supplementary section 2). This ensured that the selection criteria could be applied independently by two reviewers (DW and CC) without the risk of the websites returned altering.

### Selection of sources of evidence

**Figure 1.**
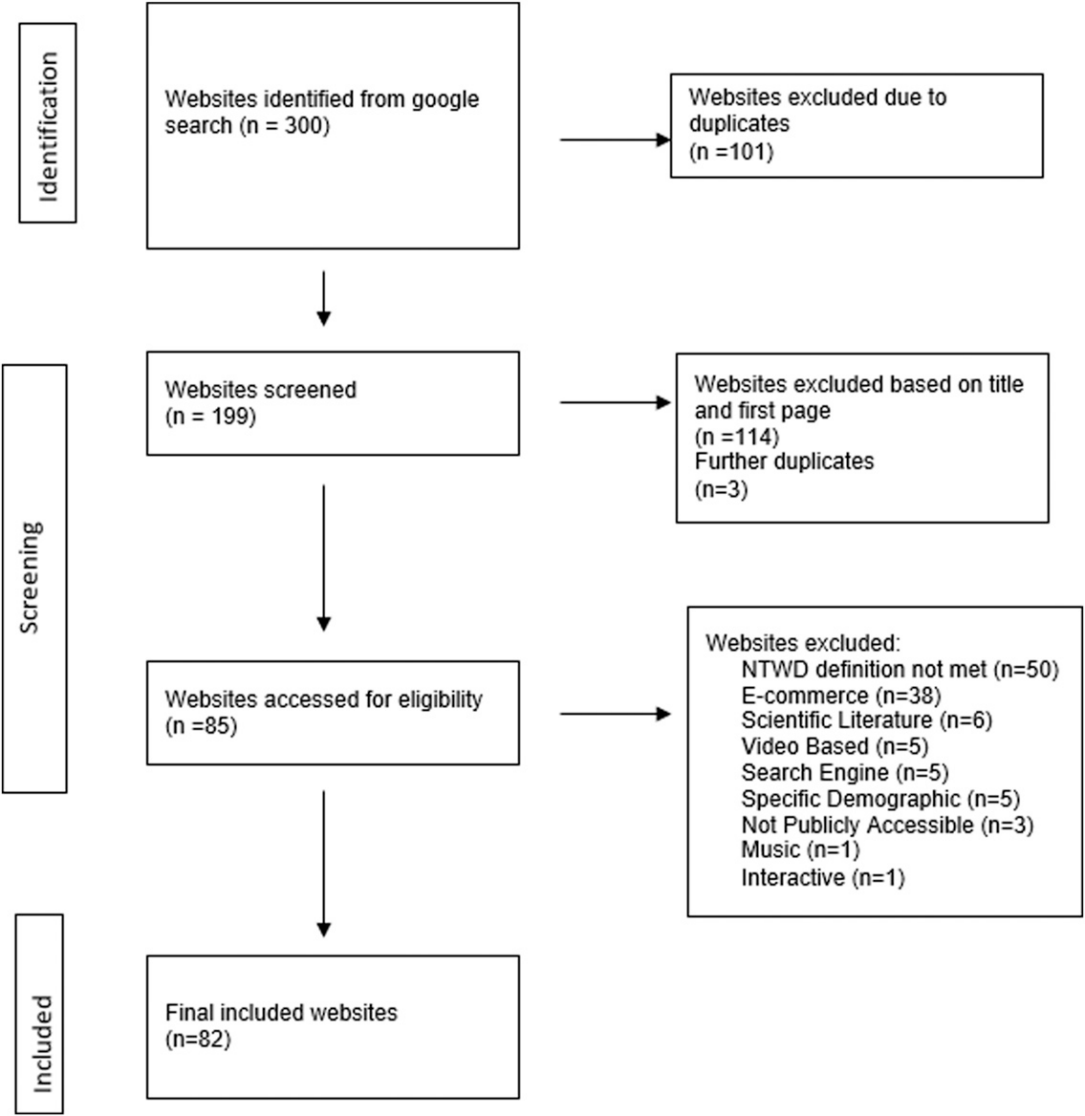
PRISMA flowchart outlining the selection process of websites for inclusion in the scoping review of NTWD websites. The search strategy returned a total of 300 websites of which 101 were removed as duplicates. Eighty-two websites met inclusion criteria (Figure 1).

Websites were screened for inclusion in the study by clicking through and reading the first page to ensure eligibility criteria were met. The decision whether the website was included or excluded and the reason for this was recorded on a spreadsheet (Supplementary section 2). This process was conducted independently by two researchers (DW and CC). Following the initial screening, a third researcher (MB) independently reviewed the selected websites and made a final decision where there was no agreement. Details of the entire search process and yields are recorded in Figure 1. The final list of website references was reviewed by the principal investigator to ensure the list was accurate and complete (Table 1).

**Table 1.** General website characteristics.

Website characteristics		*N* = 82	%
Geographic origin			
USA	32–82	53	65.6
UK	83–109	26	31.7
Republic of Ireland	110	1	1.2
Australia	111	1	1.2
Canada	112	1	1.2
Funding source			
Private service	32–35,44,46–49,51,52,54,55,57,60,66–69,71,72,74–77,79,80,89,91,91,92,97–99,104,109,110	37	45.1
Local NHS trust	83,88,90,93,94,96,101–104,106,108	12	14.6
Clinical Website	36–38,43,53,56,59,64,73,85,100,113	11	13.4
Wellbeing/lifestyle magazine article	41,63,73,82,87	5	6.1
Private blog	40,58,78,87,111	5	6.1
Product sales company	42,86,95,105	4	4.9
Online encyclopaedia	50	1	1.2
Charity	84	1	1.2
NTWD condition mentioned			
Osteoarthritis	32,33,35,42,49–55,61–69,71,72,76,79,84–86,95,98,109,111	31	37.8
Ganglion	33,35,42,49–51,53,55,61–63,65–70,80,85,97–100,105,109–111	27	32.9
Sprain	43–45,47,48,56,57,63,65,81,88–90,93,94,103,108,110,113	19	23.2
De Quervain’s	32,35,37,46,49,50,55,62,64,69,70,76,77,81,97,99,100,110	18	22
Tendonitis	42,45,46,50,52,62,63,65,66,71,73,74,76,78,99,109,112	17	20.7
Ulnar sided	37,38,50,59,68,76,99,107,109,112	10	12.2
Strain	44,47,48,53,67,86,90–92,94	10	12.2
General wrist pain	58,59,82,83,96,100–102,106	9	11
Wrist weakness	34,36,39–41,87	6	7.3
Soft tissue injury	49,50,95,104,107	5	6.1
DRUJ Tear	59,99,107,112	4	4.9
Avascular Necrosis	35	1	1.2
Treatment modality recommended			
Splinting	33,35,37,42,44,44,45,49,50,52–54,54–56,59,60,63–65,67,68,70,73,74,76–79,81–83,85,86,92,94,95,98–102,105,106,108–110,110,112,113	51	62.2
Ice/cold therapy	32,35,42–46,49,50,52–54,56,57,59,60,62,63,65,66,73,75–80,82,83,86,88–95,97,99,101–106,110,110,113	50	61
Non-steroidal anti-inflammatory drugs	32,35,37,38,43–46,50,52,52–54,54,56,59,62–66,68,70,73,76–79,82–85,88,90,91,93,95–98,100–102,107–110,112,113	49	59.8
Exercise	33–43,45,52–54,58,62–65,67,73,75,80,82–88,91,91,93–96,99,101,104,106,109,110,112	44	53.7
Surgical procedure	35,37,38,42,47,49,50,52–54,54,59,60,62–68,70,71,73,79,81,85,98,100,102,107,109,112	32	39
Rest	32,35,42–46,52–54,56,62,63,73,75–79,83,86,88,92–95,99,101–103,105,110,113	31	37
Steroid injection	43,45,46,49,50,53,54,59,60,62–68,76–79,81,100–102,109,112	25	30.5
Compression	32,35,50,53,56,57,63,66,67,73,86,90,91,96,97,99,102,104,105	19	23.2
Activity modification	42,45,50,59,62,64,65,68,75,77,80,82,83,100–102,107,109,110,112,113	21	25.6
Physiotherapy	42,43,49,50,52–54,56,60,64,65,71,74,76,77,90,92,112	18	22
Elevation	32,45,46,53,56,57,89–91,96,97,99,102,104,105,108	16	19.5
Heat	44,57,64,65,67,77,79–84,101	13	15.9
Occupational therapy	46,48–50,52–54,66,71,77,78	11	13.4
Positional advice	46,52,54,58,73,75,81,82,101,110,113	11	13.4
Hand therapy	32,46,48,67,68,80,98,107,109	9	11
Manual therapy	33,48,72,80,82,91,92	7	8.5
Plasma injection	45,45,102,109	4	4.9
Taping	72,92	2	2.4
Shockwave	45,102	2	2.4
Additional data			
Frequency of treatment detailed	45,72,74–80,109–111	11	13.4
Duration of treatment detailed	33–36,38–41,85–87	11	13.4
Expected timeframe of recovery	45,66–72,74–80,83,98,109–111	17	20.7
Global health markers referred to	72–76,76–78,80,109–111	11	13.4
References provided	33–44,84–92	21	25.6
Date of last update listed	36–47,50,62,78,84,86–88,90–97,110,110	33	40.1

### Data extraction

The 16-stage DISCERN tool was applied to information from website resources as reviewers ‘read’ website text. The DISCERN tool is a validated instrument for judging the quality of written consumer health information on treatment choices (Table 2) ^21^ and has been used in online health information assessment.^19,30,31^ A DISCERN score of 1 suggests there are serious shortcomings in the information, a score of 3 suggests there is a moderate level of shortcomings but not serious, and a score of 5 would suggest minimal shortcomings. Additional data regarding website country of origin, funding source, recommended management adjuncts and their parameters, information regarding expected timescales for recovery, the presence of references from published research, mention of global health markers and date of last update were gathered (Supplementary secton 2). Categories for funding streams of the websites were split into eight categories: private service (includes private practice and private hospital), charity (not-for-profit or condition special interest group, user led), clinical website (special interest group, professional-led), local National Health Services (NHS) trust, online encyclopaedia, private blog, product sales company and those which were pragmatically categorised as wellbeing/lifestyle magazine articles.

**Table 2.** Individual and collated DISCERN score for websites.

	Questions from DISCERN tool, measured on a 5-point Likert scale, 5 = Yes, 3 = Partially, 1 = No															
Website URL	Are the aims clear?	Does it achieve its aims?	Is it relevant?	Is it clear what sources of information have been used?	Is it clear when the information reported was produced?	Is it balanced and unbiased?	Does it provide details of additional sources of support and information?	Does it refer to areas of uncertainty?	Does it describe how each treatment works?	Does it describe the benefits of each treatment?	Does it describe the risks of each treatment?	Does it describe what would happen if no treatment was used?	Does it describe how treatment choices affect quality of life?	Is it clear there may be more than one possible treatment? Choice?	Does it provide support for shared decision making?	Collated score of on DISCERN questions
Average score	4	4	4	2	3	3	3	2	3	2	2	2	1	3	2	2.6
https://eastendot.com/what-can-i-do-for-weak-wrists-occupational-therapy/	4	3	3	2	2	2	2	1	3	3	1	2	1	3	2	2.3
https://seriouslystrongtraining.com/6-tips-to-strengthen-weak-painful-wrists/	4	3	4	1	2	2	1	1	2	2	1	1	1	1	1	1.8
https://www.medicalnewstoday.com/articles/hand-weakness	5	5	5	3	3	4	4	3	3	2	1	2	1	4	1	3.1
https://www.wristsupports.co.uk/blog/what-is-general-wrist-weakness.html	4	4	4	1	3	2	3	2	3	3	1	2	1	3	1	2.4
https://my.clevelandclinic.org/health/symptoms/17667-wrist-pain	4	4	5	3	2	4	4	2	2	1	1	2	1	5	2	2.8
https://tommorrison.uk/blog/fix-your-weak-wrists	3	3	3	1	1	2	1	1	1	2	1	1	1	2	1	1.8
https://www.healthline.com/health/how-to-strengthen-wrists	5	5	4	3	4	3	3	2	3	2	2	1	1	4	2	2.9
https://www.buoyhealth.com/learn/hand-weakness	4	5	4	4	4	4	4	3	3	3	1	2	2	4	1	3.3
https://orthoinfo.aaos.org/en/diseases--conditions/ulnar-tunnel-syndrome-of-the-wrist/	4	5	4	3	3	3	4	4	4	3	4	1	1	3	2	3.2
https://experiencelife.lifetime.life/article/fitness-fix-help-for-weak-wrists/	4	3	3	3	2	3	1	1	3	2	1	1	1	2	1	2.0
https://www.beachbodyondemand.com/blog/modified-exercises-for-weak-wrists	4	4	4	2	3	3	2	3	3	2	1	1	1	2	1	2.4
https://www.verywellhealth.com/wrist-strengthening-exercises-2696622	4	4	4	3	4	4	3	3	3	2	2	1	1	3	1	2.8
https://medi-dyne.com/pages/injury-treatment-wrist-pain	3	3	3	1	1	1	2	2	2	2	1	1	1	4	1	2.0
https://www.versusarthritis.org/about-arthritis/conditions/osteoarthritis-of-the-hand-and-wrist/	5	5	5	2	2	4	3	3	3	3	2	2	3	4	2	3.2
https://sportsmedicine.mayoclinic.org/condition/wrist-sprains/	4	3	4	1	1	3	1	1	2	1	1	1	1	3	1	1.9
https://www.mtw.nhs.uk/wp-content/uploads/2015/11/Leaflet-wrist-sprain.pdf	5	5	5	2	1	4	2	4	3	2	3	3	1	3	3	3.0
https://www.mskdiagnostics.co.uk/patients/resources/hand-and-wrist/wrist-sprain/	4	4	4	1	1	3	1	2	3	2	2	1	1	3	2	2.4
https://www.bonsecours.com/health-care-services/orthopedics-sports-medicine/hand-wrist/conditions/hand-elbow-wrist-sprain-strain	3	3	4	1	1	3	1	2	1	1	1	2	1	3	3	2.0
https://www.nhs.uk/conditions/sprains-and-strains/	4	4	5	1	4	4	4	2	3	2	1	1	1	3	2	2.8
https://www.sportsinjuryclinic.net/sport-injuries/wrist-pain/acute-wrist-injuries/wrist-strain	4	3	3	1	2	2	2	1	2	2	1	1	1	3	2	2.2
https://www.physio.co.uk/what-we-treat/musculoskeletal/conditions/wrist/wrist-strain.php	4	4	4	1	1	2	3	1	4	3	1	2	2	5	2	2.6
https://www.sports-health.com/sports-injuries/hand-and-wrist-injuries/wrist-tendonitis-vs-sprain	5	5	5	4	3	3	4	3	1	1	1	2	1	2	2	2.7
https://www.bsuh.nhs.uk/wp-content/uploads/sites/5/2016/09/Wrist-sprain.pdf	5	5	5	1	4	4	1	3	3	3	2	3	1	4	2	3.0
https://nyulangone.org/conditions/wrist-hand-repetitive-use-injuries/diagnosis	4	4	4	1	1	3	3	2	3	3	1	2	1	4	2	2.6
https://www.guysandstthomas.nhs.uk/health-information/wrist-sprains-or-strains	4	3	3	2	3	3	2	2	1	1	1	1	1	3	2	2.2
https://www.handsurgerydenver.com/wrist-sprain-vs-wrist-strain-whats-the-difference/	4	3	4	1	1	2	2	1	1	1	1	1	1	2	1	1.9
https://professionalcarept.com/have-you-sustained-a-wrist-sprain-or-strain-a-hand-therapist-could-help/	5	4	4	3	2	2	3	3	1	2	1	2	3	3	2	2.5
https://www.voltarol.co.uk/pain-treatments/wrist-pain/	4	4	4	2	1	2	2	2	2	2	1	2	2	3	2	2.5
https://www.aurorahealthcare.org/services/orthopedics/conditions/wrist-pain	4	4	3	1	1	3	2	2	3	3	1	2	1	4	3	2.4
https://en.wikipedia.org/wiki/Wrist_pain	3	3	3	2	3	2	4	1	2	2	2	2	1	2	2	2.3
https://www.nuffieldhealth.com/symptoms/hand-wrist-pain	2	3	2	1	1	3	3	2	3	3	2	3	2	4	2	2.3
https://www.merseycare.nhs.uk/hand-and-wrist-pain	4	3	4	1	1	3	2	1	3	2	2	3	3	3	2	2.4
https://www.njpaindoc.com/blog/7-causes-of-wrist-pain-you-should-never-ignore	4	4	3	2	1	2	2	2	1	1	1	1	1	1	2	1.8
https://castleortho.co.uk/hand-wrist/wrist-pain/	3	4	4	2	1	3	2	2	2	2	1	1	1	3	2	2.2
https://www.sportsinjuryclinic.net/sport-injuries/wrist-pain-by-location	4	4	4	1	2	3	4	2	2	2	1	2	1	3	2	2.5
https://healthcare.utah.edu/orthopaedics/specialties/hand-pain/when-to-see-a-doctor.php	4	4	3	1	1	2	4	2	3	2	1	2	1	2	2	2.3
https://gpnotebook.com/en-gb/simplepage.cfm?ID=624230416	3	3	3	2	3	2	3	2	1	1	1	2	1	2	1	2.0
https://www.dignityhealth.org/conditions-and-treatments/orthopedics/common-wrist-injuries-and-conditions/wrist-pain	4	4	3	1	1	2	2	2	2	2	2	2	1	3	3	2.3
https://www.newcastle-hospitals.nhs.uk/services/newcastle-occupational-health-service/physiotherapy/wrist-pain/	5	5	5	2	4	4	3	4	4	4	4	3	2	5	4	3.9
https://www.nhs.uk/conditions/hand-pain/wrist-pain/	5	4	5	2	2	4	4	4	3	3	2	2	1	4	3	3.2
https://medlineplus.gov/wristinjuriesanddisorders.html	4	4	4	3	3	4	5	2	2	2	2	2	1	3	2	2.8
https://www.mayoclinic.org/diseases-conditions/wrist-pain/symptoms-causes/syc-20366213	4	4	4	3	2	3	4	2	4	3	1	2	1	4	3	2.9
https://www.henryford.com/blog/2020/11/wrist-injuries-and-when-to-get-help	3	3	2	3	4	2	2	2	2	2	1	1	1	2	2	2.1
https://elht.nhs.uk/application/files/2115/8860/9033/Wrist_injury_leaftlet_V4.pdf	5	5	5	2	4	4	2	3	4	3	2	3	2	3	3	3.2
https://www.emedicinehealth.com/wrist_injury/article_em.html	4	3	3	2	4	3	3	2	3	2	2	2	1	2	2	2.6
https://www.hey.nhs.uk/patient-leaflet/soft-tissue-injury-wrist-hand/	4	5	5	2	5	5	2	4	4	4	5	3	4	4	3	3.8
https://www.elastoplast.co.uk/did-you-know/sports-and-activity/wrist-pain-injuries	3	3	4	1	1	2	2	2	3	3	2	1	1	3	2	2.2
https://www.drugs.com/health-guide/wrist-sprain.html	4	4	4	2	4	3	4	2	2	2	1	2	1	3	1	2.8
https://handandwristinstitute.com/whats-better-for-a-wrist-sprain-ice-or-heat/	4	4	3	2	1	3	2	3	3	3	2	1	1	3	2	2.5
https://www.gloshospitals.nhs.uk/documents/8047/Soft_tissue_injuries_affecting_the_wrist_and_hand_GHPI0878_04_19_p0j6Hy7.pdf	5	5	5	2	4	4	4	4	4	3	3	1	1	4	3	3.4
https://www.buckinghamshirehandsurgeon.co.uk/conditions-treated/ligament-and-soft-tissue-wrist-injury/	3	4	3	1	1	2	3	2	3	3	5	1	1	4	2	2.5
https://yogainternational.com/article/view/healing-and-preventing-wrist-injuries	4	3	3	1	1	2	2	2	3	3	2	1	2	1	1	2.1
https://www.ruh.nhs.uk/patients/patient_information/ORT_057_Advice_after_a_wrist_sprain.pdf	5	5	5	1	3	3	2	4	4	3	2	4	2	4	4	3.4
https://www.sportsmedtoday.com/ulnarsided-wrist-injuries-va-51.html	5	4	4	4	2	4	2	2	2	2	2	1	2	3	2	2.6
https://www.woodlandssportsmedicine.com/blog/5-common-hand-and-wrist-injuries-among-athletes	4	4	2	1	1	2	2	1	1	1	1	1	1	3	2	1.9
https://www.mayoclinic.org/diseases-conditions/wrist-pain/symptoms-causes/syc-20366213	4	4	4	2	3	2	3	3	3	2	1	1	1	3	3	2.7
https://www.medicalnewstoday.com/articles/312070	5	5	5	3	5	3	5	3	3	3	2	2	2	5	3	3.5
https://www.verywellhealth.com/wrist-pain-causes-symptoms-and-treatments-2549458	5	5	5	3	5	4	5	4	5	3	2	2	2	5	4	4.0
https://www.webmd.com/pain-management/guide/hand-pain-causes	5	4	4	2	5	4	5	2	2	2	1	1	1	4	3	3.0
https://www.healthline.com/health/wrist-pain	5	4	4	5	5	4	5	3	3	2	2	1	2	4	3	3.5
https://www.nhsinform.scot/illnesses-and-conditions/muscle-bone-and-joints/self-management-advice/wrist-hand-and-finger-problems	5	5	5	3	5	4	5	3	4	3	2	1	4	5	4	3.9
https://southernpainclinic.com/blog/wrist-pain-causes-how-to-treat-it/?utm_source=rss&utm_medium=rss&utm_campaign=wrist-pain-causes-how-to-treat-it	4	4	3	1	5	2	4	1	2	2	1	1	1	3	2	2.4
https://www.assh.org/handcare/blog/5-causes-of-wrist-pain	4	3	3	1	4	2	3	2	2	2	3	2	1	3	3	2.6
https://www.assh.org/handcare/condition/ulnar-wrist-pain	5	4	4	1	1	2	5	4	3	3	2	1	1	2	2	2.7
https://www.urmc.rochester.edu/orthopaedics/hand-wrist/conditions.cfm	4	3	2	1	1	2	2	1	2	2	1	1	1	2	2	1.8
https://www.geisinger.org/health-and-wellness/wellness-articles/2018/10/04/20/06/dont-ignore-your-hand-and-wrist-pain-here-are-the-top-3-culprits	4	3	2	2	4	2	2	2	1	1	1	1	1	2	2	1.9
https://www.ivyrehab.com/news/common-causes-for-sharp-pain-in-wrists/	5	5	3	2	4	2	3	2	4	2	2	1	1	2	2	2.6
https://www.bordertherapy.com/why-does-my-wrist-hurt-when-i-bend-it-or-put-pressure-on-it/	4	3	2	1	1	1	4	1	4	3	2	1	1	3	2	2.2
https://handinjuries.com.au/index.php/blog1/entry/why-does-my-wrist-hurt-and-cause-me-pain	4	3	2	1	1	3	1	1	1	1	1	1	1	1	1	1.6
https://www2.hse.ie/conditions/hand-pain/wrist-pain/	5	4	4	2	4	4	3	3	1	1	2	2	1	4	2	2.8
https://www.sports-health.com/sports-injuries/hand-and-wrist-injuries/symptoms-wrist-tendonitis	5	4	4	4	4	4	4	4	4	4	2	1	2	4	3	3.5
https://adventpt.com/top-of-wrist-pain-it-might-not-be-carpal-tunnel-syndrome/	3	4	3	1	1	2	2	1	3	2	1	2	1	3	2	2.2
https://carrolltonortho.com/news-events/posts/wrist/8-ways-to-easily-treat-and-prevent-wrist-pain/	4	3	4	1	3	2	2	1	3	3	1	1	1	3	2	2.4
https://newyorkhandandnerve.com/services/hand-upper-extremity/causes-hand-wrist-pain/	4	5	4	4	2	2	3	2	2	2	1	2	1	4	3	2.7
https://familydoctor.org/condition/de-quervains-tenosynovitis/	4	4	3	2	2	3	3	2	2	2	1	1	1	3	3	2.4
https://www.henryford.com/blog/2017/10/carpal-tunnel-vs-tendonitis-identifying-symptoms	5	5	4	3	4	4	3	3	2	2	2	2	2	4	3	3.1
https://medicine.umich.edu/dept/orthopaedic-surgery/patient-care-services-hand-upper-extremity/post-traumatic-wrist-arthritis	3	3	3	2	2	2	2	4	3	3	2	2	2	4	3	2.6
https://sportdoctorlondon.com/wrist-pain-and-popping/	4	4	4	2	4	3	5	3	2	2	1	1	1	3	3	2.7
https://twinboro.com/why-does-my-wrist-hurt	4	4	4	1	2	3	3	1	3	3	1	1	2	4	3	2.5
https://yorkvillephysiotherapy.com/why-does-the-pinky-side-of-my-wrist-hurt/	5	4	4	5	1	3	4	3	3	2	1	1	2	4	3	3.0
https://www.buoyhealth.com/learn/wrist-pain	5	4	4	4	4	4	4	2	2	3	2	1	2	4	4	3.2
https://www.wikihow.com/Relieve-Wrist-Pain-from-Lifting	5	5	5	3	4	5	2	2	5	3	3	2	1	4	4	3.5

Results were charted on an Excel data extraction document (Supplementary Section 2). Data extraction was independently trialled by DW and CC for the first five included websites to assess the suitability and ensure all relevant information was obtained to address the scoping review objectives. No changes were required to the charting table. DW and CC were responsible for extracting the data independently while a third reviewer (MB) arbitrated in the event of a disagreement.

## Results

Websites included in the review originated most often from USA (*n* = 53), and UK (*n* = 26), of which 50% were derived from NHS trusts. The largest funding group for public-facing websites was private health service providers (37 websites, 45.1%), followed by local NHS trusts (12 websites, 14.6%) then professional-led clinical websites (11 websites, 13.4%).

There were 12 terms found to describe NTWDs of 7 referred to lesion-based diagnoses with osteoarthritis, ganglion, De Quervain’s tenosynovitis and tendonitis being the most frequently used. When websites did not explicitly name a disorder, they referred to broader symptom-based classifications such as ‘generalised wrist pain’, ‘wrist weakness’, ‘sprain’, ‘strain’ and ‘soft-tissue injury’.

20 different treatment suggestions were found in the website material. The three most commonly recommended treatments were classified passively applied modalities: splinting (51 websites, 62.2%), ice/cold therapy (50 websites, 61%), non-steroidal anti-inflammatory medication (49 websites, 59.8%). A minority of websites detailed the parameters of how frequently the interventions were to be applied, and for how long they should be administered for (11 websites 13.4% respectively). Exercise was suggested as the fourth most common treatment modality however great variety was observed in its presentation, some websites using detailed images and written instruction while others recommended generic wrist movement without advice on dosage. More invasive management including surgery (32 websites, 39%) and cortisone injections (25 websites, 30.5%) were commonly recommended. A number of websites referred to occupational therapy, hand therapy and physiotherapy as treatments rather than professionals who may administer management strategies.

Data extracted about the presence of references and date of update were found in 21 (25.6%) and 33 (40.1%) of websites respectively.

**Figure 2.**
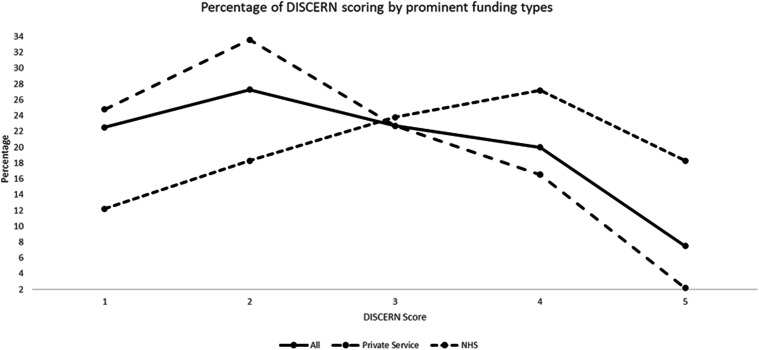
Chart to show the spread of DISCERN scores for all websites and for private and NHS services.

By grouping the websites into sources of funding, we found that there was generally better quality of resource presented on NHS websites compared to all website information and private service information (Figure 2). Raw data for all funding sources can be found in Supplementary section 2.

Of the websites produced in the UK, 50% were created by regional NHS trusts and typically provided education about the condition itself, strategies for self-management, with indications when to seek medical help.^83,88,90,93,94,96,101–104,106,108^

## Discussion

For the first time, public-facing websites for NTWDs have been identified, described and their quality assessed in response to mixed stakeholder group questions.^9^ Our scoping review found that most website resources returned through a search which aimed to replicate a British lay-persons online behaviour, originated not in the UK, but in the USA. The country of origin or source of funding was often not immediately obvious when accessing the websites, which may cause confusion for UK-based online health seekers and set expectations for access to management options which may not be available through local health services.

Lesion-based NTWDs such as osteoarthritis, ganglion, de Quervain’s and tendonitis were found frequently despite poor evidence for a) accurate lesion-specific diagnosis or b) that diagnostic specificity changes management strategies and explains the extent of pain and disability experienced.^10–15^ We observed passive modalities (splinting, ice and non-steroidal anti-inflammatories) were most commonly recommended, followed by exercise. Minimal guidance was given about the frequency or duration of recommended interventions, suggesting a knowledge gap. Another gap noted was information on any impact of global health markers on NTWDs as this was seldom referenced. The large variety of conservative and non-conservative interventions suggested across the websites may reflect the lack of evidence demonstrating superiority of one treatment over another, as well as the dearth of high quality guidelines for best practice.^9,18^ It is notable that likely timescales for normal recovery were only given in 20% of sources, suggesting a lack of understanding about natural history of NTWDs or an assumption this information is not desired by those with NTWD. Given the above, it is likely to be difficult for online health seekers to become evidence-informed about their healthcare options and to navigate toward best care. The range of quality scores between different NHS websites demonstrates variability within the UK public sector and reinforces the need for agreed standards of care through best-practice guidelines specific to the UK public healthcare system. Special interest groups, NHS clinical leadership organisations and peer-led charities may be well placed to develop these.

Differences were observed in the weighting of content provided by websites based on location and funding source. Although NHS trusts websites provided some information regarding more invasive interventions, USA-based private health-service websites appeared to devote more resource to surgical interventions. Similarly, websites advertising private healthcare had greater prominence of more invasive treatments whereas not-for-profit websites (NHS, charities and clinical websites) were more likely to promote conservative care and self-management. For online health seekers, the weighting of management options may have implications on their decision-making for care, especially given the poor scores for questions about whether the websites promoted shared decision making, whether benefits or risks of interventions were clearly stated and how treatment choices may affect quality of life found using the DISCERN tool. The consideration that many people lack the capability to recognise the strengths, weaknesses or credibility of the information presented on websites^21^ add to concerns over the impact of poor quality information on individuals healthcare decision-making and highlights the need for trustworthy online resources.^114^

Consistent with previous studies examining public-facing, online health-related information,^115,116^ websites from non-profit organisations were found to be higher quality information sources than sales-focused websites. The authors recommend more clarity of specific parameters for recommended interventions, such as adherence, frequency or specific exercises based on experimental research would help support guidelines for management.

### Strengths and limitations of the review

The strengths of this study included pre-registration on the Open Science Framework, use of a novel search strategy, mapping data to key stakeholder questions, and adherence to PRISMA ScR guidelines. Limitations included a lack of regard to different information modalities and only including websites written in English. Video items hosted on social media and other platforms are becoming a greater source of information for online health seekers, particularly for younger age groups. These present a more complex proposition for data extraction and content analysis but should be considered in future work. Performing a reflexive thematic analysis as intended in the proposal may have allowed greater value in our exploration of the data through understanding the impact of the researcher’s subjectivity and biases.

## Conclusion

Public facing websites for NTWD show considerable variation in the information presented. The quality and trustworthiness of website information on NTWD are not the preserve of any sector or organisation and we identified potential for improvements across the board.

The majority of resources for NTWD available to UK online health seekers are based in the USA. This raises particular issues on their applicability to UK healthcare and the trustworthiness of information given. Information from NHS Trusts comprise an important minority of websites, however, the quality and content of their information can be improved, and we recommend the country of origin and funding source is clearly stated.

## Supplemental Material

Supplemental Material - A scoping review to map public-facing websites for non-traumatic wrist disorders with quality evaluation Supplemental Material for A scoping review to map public-facing websites for non-traumatic wrist disorders with quality evaluation by Thomas Mitchell, Michael Bircumshaw, Clare Cryan, Dawid Kotwica, Nick Hamilton, Ben Dean, and Sionnadh McLean in Hand Therapy

Supplemental Material - A scoping review to map public-facing websites for non-traumatic wrist disorders with quality evaluation Supplemental Material for A scoping review to map public-facing websites for non-traumatic wrist disorders with quality evaluation by Thomas Mitchell, Michael Bircumshaw, Clare Cryan, Dawid Kotwica, Nick Hamilton, Ben Dean, and Sionnadh McLean in Hand Therapy
