# Supplementary material for: A scoping review to map public-facing websites for non-traumatic wrist disorders with quality evaluation
Source: Hand Ther. 2024 Oct 8;30(1):3–18. doi: 10.1177/17589983241287082 (PMC11559518; doi:10.1177/17589983241287082)
Supplement: Supplemental Material - A scoping review to map public-facing websites for non-traumatic wrist disorders with quality evaluation [file sj-pdf-1-hth-10.1177_17589983241287082.pdf]

Supplementary section 1. Lay terms used for Google^{TM} search derived from mixed stakeholder group (1)

- Weak wrist
- Wrist sprain
- Wrist strain
- Wrist pain
- Wrist injury
- Why does my wrist hurt

---

1. Mitchell T, Hamilton N, Dean B, Rodgers S, Fowler-Davis S, McLean S. A scoping review to map evidence regarding key domains and questions in the management of non-traumatic wrist disorders. Hand Therapy. 2023 Dec 12;17589983231219595.
