# Supplementary material for: A scoping review to map public-facing websites for non-traumatic wrist disorders with quality evaluation
Source: Hand Ther. 2024 Oct 8;30(1):3–18. doi: 10.1177/17589983241287082 (PMC11559518; doi:10.1177/17589983241287082)
Supplement: Supplemental Material - A scoping review to map public-facing websites for non-traumatic wrist disorders with quality evaluation [file sj-pdf-2-hth-10.1177_17589983241287082.pdf]

Supplementary section 2.

Search results with inclusion and exclusion reasoning

			DW		CC				MB	
Number	Search Term	URL	Included	Reason	Included	Reason	Discussion	CW and DK Comments	Included	Reason
1	Weak Wrist	https://www.nhs.uk/conditions/hand-pain/wrist-pain/	NO	Duplicate	NO	Duplicate	NO		NO	Duplicate
2	Weak Wrist	https://www.mayoclinic.org/diseases-conditions/wrist-pain/symptoms-causes/syc-20366213	NO	Duplicate	NO	Duplicate	NO		NO	Duplicate
3	Weak Wrist	https://eastendot.com/what-can-i-do-for-weak-wrists-occupational-therapy/	YES	Included Cyst info interchangeable	YES		YES		YES	
4	Weak Wrist	https://www.nhsinform.scot/illnesses-and-conditions/muscle-bone-and-joints/exercises/exercises-for-wrist-hand-and-finger-problems	NO	Video content avaleable NO NTWD umbrella term mentioned	YES	Generic wrist info in text	MB?		NO	Video
5	Weak Wrist	https://seriouslystrongtraining.com/6-tips-to-strengthen-weak-painful-wrists/	NO	NO NTWD terms included	YES	advice	YES	MOWP - other "unkown"	YES	
6	Weak Wrist	https://www.medicalnewstoday.com/articles/hand-weakness	YES	Included Cyst info interchangeable	YES		YES		YES	
7	Weak Wrist	https://www.wristsupports.co.uk/blog/what-is-general-wrist-weakness.html	NO	NO NTWD terms included	YES		YES	MOWP - other "unkown"	YES	
8	Weak Wrist	https://my.clevelandclinic.org/health/symptoms/17667-wrist-pain	YES	Included Cyst info interchangeable	YES		YES		YES	
9	Weak Wrist	https://tommorrison.uk/blog/fix-your-weak-wrists	NO	mentioned	YES	Generic Wrist	MB?	Videos inc + text	YES	text hybrid
10	Weak Wrist	https://www.healthline.com/health/how-to-strengthen-wrists	NO	NO NTWD terms included + Strengthening only rather that PAIN related terms	YES	General Wrist ex	MB?		YES	
11	Weak Wrist	https://handexer.com/blogs/grip-strength-and-hand-health/common-causes-of-wrist-and-hand-weakness	NO	No NTWD terms mentioned	YES		MB?	Weak info on NTWD	NO	Not NTWD
12	Weak Wrist	https://www.buoyhealth.com/learn/hand-weakness	NO	No NTWD terms	YES		YES		YES	
13	Weak Wrist	https://www.youtube.com/watch?v=kdz8L5g32Vw	NO	Youtube	NO	Youtube			NO	Video
14	Weak Wrist	https://www.arthritis.org/health-wellness/about-arthritis/related-conditions/physical-effects/expertqa-strengthen-your-wrists	NO	NO NTWD Terms	YES		MB?		NO	Not NTWD
15	Weak Wrist	https://www.arthritis.org/health-wellness/about-arthritis/where-it-hurts/when-hand-or-wrist-pain-may-mean-arthritis	NO	Duplicate	NO	Duplicate			NO	Duplicate
16	Weak Wrist	https://orthoinfo.aaos.org/en/diseases--conditions/ulnar-tunnel-syndrome-of-the-wrist/	YES	Included Cyst info interchangeable	YES				YES	
17	Weak Wrist	https://www.urmc.rochester.edu/orthopaedics/hand-wrist/conditions.cfm	NO	Duplicate	NO	Duplicate			NO	Duplicate
18	Weak Wrist	https://medlineplus.gov/ency/article/003175.htm	NO	NO NTWD terms	YES				NO	Not NTWD
19	Weak Wrist	https://hordercentre.co.uk/news/hand-therapy-qa-what-is-causing-my-wrist-pain-at-the-gym/	NO	NO NTWD terms	YES	advice	MB?		NO	Not NTWD
20	Weak Wrist	https://www.elastoplast.co.uk/products/muscle-joint-and-back-pain/protective-wrist-support?gclid=EAIaIQobChMI7cyN2Jrg_QIVIrPtCh	NO	AD	NO	Ad			NO	Ad
21	Weak Wrist	https://www.physiocheck.co.uk/check/start?vari=1&gclid=EAIaIQobChMI7cyN2Jrg_QIVjrPtCh18mAEDEAMYAiAAEgLbofD_BwE	NO	Symptom Checker	NO	Checker			NO	Interactive
22	Weak Wrist	https://www.amazon.co.uk/s?k=weak+wrist&adgrpid=61753293068&hvadid=259057049757&hvdev=c&hvlocphy=9046357&hvnetw=g&	NO	AD	NO	Ad			NO	Ad
23	Weak Wrist	https://www.googleadservices.com/pagead/aclk?sa=L&ai=DChcSEwic_tqEnOD9AhWn7-0KHbACDg0YABAEGgJkZw&ohost=www.go	NO	AD	NO	Ad			NO	Ad
24	Weak Wrist	https://www.search-hq.com/web?q=best%20treatment%20for%20wrist%20pain&kwid=best%20treatment%20for%20wrist%20pain&cl	NO	Search Engine	NO	Engine			NO	Search Engine
25	Weak Wrist	https://injury-specialists-direct.com/sip/wrist-injury/?src=google&kw=pain%20in%20wristb&gclid=EAIaIQobChMI3P7ahJzg_QIVp-_tCh	NO	AD	NO	Ad			NO	Ad
26	Weak Wrist	https://www.esht.nhs.uk/wp-content/uploads/2017/06/0476.pdf	NO	Wrist Fracture	NO	Wrist fracture			NO	Not NTWD
27	Weak Wrist	https://journals.lww.com/ajpmr/Fulltext/2021/02000/Two_Years_of_Progressive_Right_Wrist_Pain_and_Hand.18.aspx	NO	Journal	NO	Journal			NO	Journal
28	Weak Wrist	https://www.hopkinsmedicine.org/health/conditions-and-diseases/lateral-epicondylitis-tennis-elbow	NO	NO NTWD	NO	NO NTWD			NO	Not NTWD
29	Weak Wrist	https://experiencelife.lifetime.life/article/fitness-fix-help-for-weak-wrists/	YES	Included	YES				YES	
30	Weak Wrist	https://www.beachbodyondemand.com/blog/modified-exercises-for-weak-wrists	YES	Included	YES				YES	
31	Weak Wrist	https://www.hss.edu/golfportal/tips-for-protecting-your-wrist-on-the-golf-course.htm	NO	Not created for NTWD	YES		MB?	Sport specific?	NO	Not NTWD
32	Weak Wrist	https://www.verywellhealth.com/wrist-strengthening-exercises-2696622	NO	NO NTWD	YES		MB?	generic strengthening	YES	
33	Weak Wrist	https://www.ninds.nih.gov/health-information/disorders/carpal-tunnel-syndrome	NO	Duplicate	NO	Duplicate			NO	Duplicate
34	Weak Wrist	https://www.ncbi.nlm.nih.gov/books/NBK532993/	NO	Journal	NO	Journal			NO	Journal
35	Weak Wrist	https://www.amazon.co.uk/Neo-Wrist-Band-Instability-Compression/dp/B00WUK7IVM	NO	AD	NO	Ad			NO	Ad
36	Weak Wrist	https://www.pematon.co.uk/?gclid=EAIaIQobChMI3P7ahJzg_QIVp-_tCh2wAg6tEAMYASAAEgKpivD_BwE	NO	AD	NO	Ad			NO	Ad
37	Weak Wrist	https://s.healthnwell.com/serp?q=how%20to%20treat%20wrist%20pain&segment=healthnwell.g016&s1aid=8859207431&s1cid=1579	NO	Search Engine	NO	Search engine			NO	Search Engine
38	Weak Wrist	https://www.izito.ws/ws?q=pain%20relief%20for%20wrist%20pain&asid=iz_ws_gc1_02&abt=1&mt=b&nw=g&de=c&ap=&kid=kwd-511	NO	Search Engine	NO	Search engine			NO	Search Engine
39	Weak Wrist	https://www.dystonia.org.uk/Pages/FAQs/Category/hand-dystonia	NO	NO NTWD	NO	NO NTWD			NO	Not NTWD
40	Weak Wrist	https://medi-dyne.com/pages/injury-treatment-wrist-pain	YES	Included Cyst info interchangeable	YES				YES	
41	Weak Wrist	https://www.mskdorset.nhs.uk/hand-and-wrist-pain/	NO	NO NTWD	YES		MB?	1st page limited info	NO	Not NTWD
42	Weak Wrist	http://www.easywellbeing.com/default/oppo-2181-elastic-wrist-wrap-support-weak-carpal-tunnel-tendon-sprain-brace-nhs.html	NO	AD	NO	Ad			NO	Ad
43	Weak Wrist	https://www2.hse.ie/conditions/thumb-wrist-pain-after-baby/	NO	NO NTWD	YES		MB?	Condition specific - pregn	NO	Specific Demographic
44	Weak Wrist	https://www.versusarthritis.org/about-arthritis/conditions/osteoarthritis-of-the-hand-and-wrist/	YES	Generic OA context	NO	Base of thumb/RA	Yes	generic OA and ex	YES	
45	Weak Wrist	https://www.britishcycling.org.uk/knowledge/training/health-recovery/article/izn20140820-Physiotherapy-Ask-the-Experts--Hand-and-w	NO	Cycling Specific	NO	cycling specific			NO	Specific Demographic
46	Weak Wrist	https://www.wihb.scot.nhs.uk/wp-content/uploads/2020/03/Getting-your-wrist-moving-after-your-cast-is-removed-1-1.pdf	NO	NO NTWD	NO	No NTWD			NO	Not NTWD
47	Weak Wrist	https://weakwrists.bandcamp.com/album/weak-wrists	NO	Music	NO	Music			NO	Music
48	Weak Wrist	https://www.uhcw.nhs.uk/download/clientfiles/files/Patient%20Information%20Leaflets/Clinical%20Support%20Services/Physiotherapy	NO	NO NTWD	NO	No NTWD			NO	Not NTWD
49	Weak Wrist	https://www.assh.org/handcare/condition/cubital-tunnel-syndrome	NO	NO NTWD	NO	No NTWD			NO	Not NTWD
50	Weak Wrist	https://signatureclinic.co.uk/carpal-tunnel-decompression-carpal-tunnel-syndrome-signature-clinic-uk/?gclid=EAIaIQobChMIuNO7qp3	NO	NO NTWD	NO	No NTWD			NO	Not NTWD
1	Wrist Sprain	https://www.voltarol.co.uk/understanding-pain/pain-types/?gclid=EAIaIQobChMIpOOPtJ7g_QIVD9PtCh2CQQMIEAAYASAAEgLK6fD_	NO	Duplicate	NO	Duplicate			NO	Duplicate
2	Wrist Sprain	https://www.ibuleve.com/products?gclid=EAIaIQobChMIpOOPtJ7g_QIVD9PtCh2CQQMIEAAYAiAAEgIpXPD_BwE&gclid=EAIaIQobCh	NO	Duplicate	NO	Duplicate			NO	Duplicate
3	Wrist Sprain	https://www.amazon.co.uk/s?k=wrist+sprain&adgrpid=55989016074&hvadid=578374821246&hvdev=c&hvlocphy=1007064&hvnetw=g	NO	AD	NO	Duplicate			NO	Duplicate
4	Wrist Sprain	https://orthoinfo.aaos.org/en/diseases--conditions/wrist-sprains/	NO	NO NTWD	YES	Sprains	MB?	MOWP doesn't specify	NO	Not NTWD
5	Wrist Sprain	https://www.webmd.com/fitness-exercise/wrist-sprain	NO	Duplicate	NO	Duplicate			NO	Duplicate
6	Wrist Sprain	https://www.nhs.uk/conditions/sprains-and-strains/	NO	Duplicate	NO	Duplicate			NO	Duplicate
7	Wrist Sprain	https://www.assh.org/handcare/condition/sprained-wrist	NO	Duplicate	NO	Duplicate			NO	Duplicate
8	Wrist Sprain	https://my.clevelandclinic.org/health/diseases/24558-sprained-wrist	NO	NO NTWD	YES		MB?	MOWP doesn't specify	NO	Not NTWD
9	Wrist Sprain	https://myhealth.alberta.ca/Health/aftercareinformation/pages/conditions.aspx?hwid=uf7611	NO	NO NTWD	YES		MB?	MOWP doesn't specify	NO	Not NTWD
10	Wrist Sprain	https://www.physio-pedia.com/Wrist_Sprain	NO	Duplicate	NO	Duplicate			NO	Duplicate
11	Wrist Sprain	https://www.ncbi.nlm.nih.gov/books/NBK551514/	NO	Duplicate	NO	Duplicate			NO	Duplicate
12	Wrist Sprain	https://sportsmedicine.mayoclinic.org/condition/wrist-sprains/	NO	NO NTWD	YES		MB?	MOWP doesn't specify	YES	NTWD
13	Wrist Sprain	https://www.asra.com/?gclid=EAIaIQobChMIpOOPtJ7g_QIVD9PtCh2CQQMIEAMYASAAEgIEnvD_BwE	NO	AD	NO	Ad			NO	Ad
14	Wrist Sprain	https://www.zapmeta.ws/ws?q=swollen%20wrist%20treatment&asid=ws_gc14_04&abt=1&mt=b&nw=g&de=c&ap=&kid=kwd-3214997	NO	Search Engine	NO	Search			NO	Search Engine
15	Wrist Sprain	https://www.googleadservices.com/pagead/aclk?sa=L&ai=DChcSEwik44-0nuD9AhUP0-0KHYJBAyUYABABGgJkZw&ohost=www.goo	NO	NO NTWD	NO	No NTWD			NO	Not NTWD
16	Wrist Sprain	https://www.ibuleve.com/products?gclid=EAIaIQobChMIr4Op1J_g_QIVWdTtCh1kXgMcEAAYASAAEgKHufD_BwE&gclid=EAIaIQobC	NO	AD	NO	Ad			NO	Ad
17	Wrist Sprain	https://www.amazon.co.uk/s?k=wrist+sprain&adgrpid=55989016074&hvadid=578374821246&hvdev=c&hvlocphy=1007064&hvnetw=g	NO	Duplicate	NO	Duplicate			NO	Duplicate
18	Wrist Sprain	https://www.voltarol.co.uk/understanding-pain/pain-types/?gclid=EAIaIQobChMIr4Op1J_g_QIVWdTtCh1kXgMcEAAYAyAAEgJR6PD_	NO	Duplicate	NO	Duplicate			NO	Duplicate
19	Wrist Sprain	https://medlineplus.gov/ency/patientinstructions/000568.htm	NO	Duplicate	NO	Duplicate			NO	Duplicate
20	Wrist Sprain	https://handandwristinstitute.com/wrist-sprain/	NO	NO NTWD	YES		MB?	MOWP doesn't specify	NO	Not NTWD
21	Wrist Sprain	https://www.nationwidechildrens.org/specialties/sports-medicine/sports-medicine-articles/wrist-sprains	NO	Duplicate	NO	Duplicate			NO	Duplicate

22	Wrist Sprain	https://onlinepare.net/dashboard.php	NO	Non publucally accesible	NO	Not publically accesible				NO	Not publicaly Accessible
23	Wrist Sprain	https://www.sports-health.com/sports-injuries/hand-and-wrist-injuries/symptoms-wrist-sprain	NO	Duplicate	NO	Duplicate				NO	Duplicate
24	Wrist Sprain	https://www.guysandstthomas.nhs.uk/health-information/wrist-sprains-or-strains	NO	Duplicate	NO	Duplicate				NO	Duplicate
25	Wrist Sprain	https://www.healthline.com/health/sprained-wrist	NO	NO NTWD	YES		MB?	MOWP doesn't specify		NO	Not NTWD
26	Wrist Sprain	http://www.mtw.nhs.uk/wp-content/uploads/2015/11/Leaflet-wrist-sprain.pdf	NO	NO NTWD	YES		MB?	MOWP doesn't specify		YES	Non
27	Wrist Sprain	https://www.uhsussex.nhs.uk/resources/wrist-sprain-the-alex/	NO	NO NTWD	YES		MB?	MOWP doesn't specify		NO	Not NTWD
28	Wrist Sprain	https://www.sportsradiology.co.uk/wrist-sprain.html	NO	NO NTWD	YES		MB?	MOWP doesn't specify		NO	Not NTWD
29	Wrist Sprain	https://www.pematon.co.uk/?gclid=EAIaIQobChMIr4Op1J_g_QIVWdTtCh1kXgMcEAMYASAAEgJeWPD_BwE	NO	Duplicate	NO	Duplicate				NO	Duplicate
30	Wrist Sprain	https://www.orthteamcentre.co.uk/?ds_rl=1292995&ds_rl=1292995&gclid=EAIaIQobChMIr4Op1J_g_QIVWdTtCh1kXgMcEAMYAiAAE	NO	AD	NO	Ad				NO	Ad
31	Wrist Sprain	https://www.elastoplast.co.uk/products/muscle-joint-and-back-pain/protective-wrist-support?gclid=EAIaIQobChMIr4Op1J_g_QIVWdTt	NO	Duplicate	NO	Duplicate				NO	Duplicate
32	Wrist Sprain	https://www.voltarol.co.uk/understanding-pain/pain-types/?gclid=EAIaIQobChMIqdnDmKHg_QIVFevtCh1hgQkbEAAYASAAEgJvkvD_	NO	Duplicate	NO	Duplicate				NO	Duplicate
33	Wrist Sprain	https://www.amazon.co.uk/s?k=wrist+sprain&adgrpid=55989016074&hvadid=578374821246&hvdev=c&hvlocphy=1007064&hvnetw=g	NO	Duplicate	NO	Duplicate				NO	Duplicate
34	Wrist Sprain	https://www.ibuleve.com/products?gclid=EAIaIQobChMIqdnDmKHg_QIVFevtCh1hgQkbEAAYAyAAEgId4vD_BwE&gclid=EAIaIQobCh	NO	Duplicate	NO	Duplicate				NO	Duplicate
35	Wrist Sprain	https://www.nationwidechildrens.org/conditions/wrist-and-hand-sprains	NO	Duplicate	NO	Duplicate				NO	Duplicate
36	Wrist Sprain	https://www.sportsinjuryclinic.net/sport-injuries/wrist-pain/acute-wrist-injuries/wrist-strain	NO	Duplicate	NO	Duplicate				NO	Duplicate
37	Wrist Sprain	https://www.massgeneralbrigham.org/en/patient-care/services-and-specialties/sports-medicine/conditions/hand-arm/wrist-sprain	NO	Duplicate	NO	Duplicate				NO	Duplicate
38	Wrist Sprain	https://www.health.harvard.edu/a_to_z/wrist-sprain-a-to-z	NO	NO NTWD	NO	No NTWD				NO	Not NTWD
39	Wrist Sprain	https://www.fairview.org/patient-education/90645	NO	Duplicate	NO	Duplicate				NO	Duplicate
40	Wrist Sprain	https://www.bouldercentre.com/news/wrist-sprain-common-injury	NO	Duplicate	NO	Duplicate				NO	Duplicate
41	Wrist Sprain	https://www.bone-joint.com/wrist-sprain-vs-wrist-strain-whats-the-difference/	NO	NO NTWD	YES		MB?	MOWP doesn't specify		NO	Not NTWD
42	Wrist Sprain	https://www.mskdiagnostics.co.uk/patients/resources/hand-and-wrist/wrist-sprain/	NO	NO NTWD	YES		MB?	MOWP doesn't specify		YES	NTWD
43	Wrist Sprain	https://www.bonsecours.com/health-care-services/orthopedics-sports-medicine/hand-wrist/conditions/hand-elbow-wrist-sprain-strain	NO	NO NTWD	YES		MB?	MOWP doesn't specify		YES	Loose wrist info - include
44	Wrist Sprain	https://www.nbt.nhs.uk/our-services/a-z-services/emergency-zone/ed-miu-patient-information/how-treat-your-injured-wrist	NO	Duplicate	NO	Duplicate				NO	Duplicate
45	Wrist Sprain	https://thegaryflemingpractice.co.uk/?gclid=EAIaIQobChMIqdnDmKHg_QIVFevtCh1hgQkbEAMYASAAEgImlfD_BwE	NO	AD	NO	Ad				NO	Ad
46	Wrist Sprain	https://www.googleadservices.com/pagead/aclk?sa=L&ai=DChcSEwip2cOYoeD9AhUV6-0KHWGBCRsYABAAGgJkZw&ohost=www.g	NO	AD	NO	Ad				NO	Ad
47	Wrist Sprain	https://injury-specialists-direct.com/sip/wrist-injury/?src=google&kw=sprained%20wristb&gclid=EAIaIQobChMIqdnDmKHg_QIVFevtCh	NO	Duplicate	NO	Duplicate				NO	Duplicate
48	Wrist Sprain	https://www.voltarol.co.uk/understanding-pain/pain-types/?gclid=EAIaIQobChMI89nl06Lg_QIVVeztCh1OdQ95EAAYASAAEgLcxvD_B	NO	AD	NO	General pain advice				NO	Not NTWD
49	Wrist Sprain	https://www.amazon.co.uk/s?k=wrist+sprain&adgrpid=55989016074&hvadid=578374821246&hvdev=c&hvlocphy=1007064&hvnetw=g	NO	Duplicate	NO	Duplicate				NO	Duplicate
50	Wrist Sprain	https://www.ibuleve.com/products?gclid=EAIaIQobChMI89nl06Lg_QIVVeztCh1OdQ95EAAYAyAAEgIrvPD_BwE&gclid=EAIaIQobChM	NO	Duplicate	NO	Duplicate				NO	Duplicate
1	Wrist Strain	https://orthoinfo.aaos.org/en/diseases--conditions/wrist-sprains/	NO	Duplicate	NO	Duplicate				NO	Duplicate
2	Wrist Strain	https://www.nhs.uk/conditions/sprains-and-strains/	NO	NO NTWD	YES		MB?	MOWP doesn't specify		YES	
3	Wrist Strain	https://www.webmd.com/fitness-exercise/wrist-sprain	NO	Duplicate	NO	Duplicate				NO	Duplicate
4	Wrist Strain	https://myhealth.alberta.ca/Health/aftercareinformation/pages/conditions.aspx?hwid=uf7611	NO	Duplicate	NO	Duplicate				NO	Duplicate
5	Wrist Strain	https://www.sportsinjuryclinic.net/sport-injuries/wrist-pain/acute-wrist-injuries/wrist-strain	NO	NO NTWD	YES		MB?	MOWP doesn't specify		YES	
6	Wrist Strain	https://www.assh.org/handcare/condition/sprained-wrist	NO	Duplicate	NO	Duplicate				NO	Duplicate
7	Wrist Strain	https://my.clevelandclinic.org/health/diseases/24558-sprained-wrist	NO	Duplicate	NO	Duplicate				NO	Duplicate
8	Wrist Strain	https://www.physio-pedia.com/Wrist_Sprain	NO	NO NTWD + Video	YES		MB?	MOWP doesn't specify + v		NO	Video
9	Wrist Strain	https://www.bone-joint.com/wrist-sprain-vs-wrist-strain-whats-the-difference/	NO	Duplicate	NO	Duplicate				NO	Duplicate
10	Wrist Strain	https://www.googleadservices.com/pagead/aclk?sa=L&ai=DChcSEwiancyhmeD9AhWk7-0KHav4BVQYABABGgJkZw&ohost=www.go	NO	AD	NO	Ad				NO	Ad
11	Wrist Strain	https://www.googleadservices.com/pagead/aclk?sa=L&ai=DChcSEwiancyhmeD9AhWk7-0KHav4BVQYABAAGgJkZw&ohost=www.go	NO	AD	NO	Ad				NO	Ad
12	Wrist Strain	https://www.ncbi.nlm.nih.gov/books/NBK551514/	NO	Duplicate	NO	Duplicate				NO	Duplicate
13	Wrist Strain	https://www.bonsecours.com/health-care-services/orthopedics-sports-medicine/hand-wrist/conditions/hand-elbow-wrist-sprain-strain	NO	Duplicate	NO	Duplicate				NO	Duplicate
14	Wrist Strain	https://sportsmedicine.mayoclinic.org/condition/wrist-sprains/	NO	Duplicate	NO	Duplicate				NO	Duplicate
15	Wrist Strain	https://www.healthline.com/health/sprained-wrist	NO	Duplicate	NO	Duplicate				NO	Duplicate
16	Wrist Strain	https://www.jointonline.net/trending/content/wrist-sprain-vs-strain	NO	NO NTWD	NO	Not accessible				NO	Not publicaly Accessible
17	Wrist Strain	https://medlineplus.gov/ency/patientinstructions/000568.htm	NO	NO NTWD	YES		MB?	MOWP doesn't specify		NO	Not NTWD
18	Wrist Strain	https://handandwristinstitute.com/wrist-sprain/	NO	Duplicate	NO	Duplicate				NO	Duplicate
19	Wrist Strain	https://www.massgeneralbrigham.org/en/patient-care/services-and-specialties/sports-medicine/conditions/hand-arm/wrist-sprain	NO	Duplicate	NO	Duplicate				NO	Duplicate
20	Wrist Strain	https://www.ruh.nhs.uk/patients/patient_information/ORT_057_Advice_after_a_wrist_sprain.pdf	NO	Duplicate	NO	Duplicate				NO	Duplicate
21	Wrist Strain	https://www.physio.co.uk/what-we-treat/musculoskeletal/conditions/wrist/wrist-strain.php	NO	NO NTWD	YES		MB?	MOWP doesn't specify		YES	Various treatment methods
22	Wrist Strain	https://www.googleadservices.com/pagead/aclk?sa=L&ai=DChcSEwiancyhmeD9AhWk7-0KHav4BVQYABADGgJkZw&ohost=www.go	NO	Duplicate	NO	Duplicate				NO	Duplicate
23	Wrist Strain	https://www.googleadservices.com/pagead/aclk?sa=L&ai=DChcSEwjancyhmeD9AhWk7-0KHav4BVQYABACGgJkZw&ohost=www.go	NO	AD	NO	Ad				NO	Ad
24	Wrist Strain	https://www.googleadservices.com/pagead/aclk?sa=L&ai=DChcSEwjh04z8muD9AhVZ6-0KHefVA9QYABAFGgJkZw&ohost=www.goo	NO	AD	NO	Ad				NO	Ad
25	Wrist Strain	https://www.googleadservices.com/pagead/aclk?sa=L&ai=DChcSEwjh04z8muD9AhVZ6-0KHefVA9QYABAEGgJkZw&ohost=www.goo	NO	AD	NO	Ad				NO	Ad
26	Wrist Strain	https://www.googleadservices.com/pagead/aclk?sa=L&ai=DChcSEwih04z8muD9AhVZ6-0KHefVA9QYABADGgJkZw&ohost=www.goo	NO	AD	NO	Ad				NO	Ad
27	Wrist Strain	https://www.mayoclinic.org/diseases-conditions/wrist-pain/symptoms-causes/syc-20366213	NO	Duplicate	NO	Duplicate				NO	Duplicate
28	Wrist Strain	https://www.sports-health.com/sports-injuries/hand-and-wrist-injuries/wrist-tendonitis-vs-sprain	NO	NO NTWD	YES		MB?	MOWP doesn't specify		YES	Non traumatic
29	Wrist Strain	https://www.sports-health.com/sports-injuries/hand-and-wrist-injuries/symptoms-wrist-sprain	NO	NO NTWD	YES		MB?	MOWP doesn't specify		YES	Non traumatic
30	Wrist Strain	https://www.nationwidechildrens.org/conditions/wrist-and-hand-sprains	NO	NO NTWD	NO	No NTWD				NO	Not NTWD
31	Wrist Strain	https://www.nationwidechildrens.org/specialties/sports-medicine/sports-medicine-articles/wrist-sprains	NO	Duplicate	NO	Duplicate				NO	Duplicate
32	Wrist Strain	https://www.ahearne.co.uk/wrist-sprains-and-strains.html	NO	NO NTWD	YES		MB?	MOWP doesn't specify		NO	Not NTWD
33	Wrist Strain	https://www.bsuh.nhs.uk/wp-content/uploads/sites/5/2016/09/Wrist-sprain.pdf	NO	NO NTWD	YES		MB?	MOWP doesn't specify		YES	
34	Wrist Strain	https://www.drugs.com/health-guide/wrist-sprain.html	NO	Duplicate	NO	Duplicate				NO	Duplicate
35	Wrist Strain	https://www.bidneedham.org/departments/orthopaedics/hand-program/most-common-hand-wrist-injuries	NO	Duplicate	NO	Duplicate				NO	Duplicate
36	Wrist Strain	https://www.fairview.org/patient-education/90645	NO	NO NTWD	YES		MB?	MOWP doesn't specify		NO	Not NTWD
37	Wrist Strain	https://www.googleadservices.com/pagead/aclk?sa=L&ai=DChcSEwih04z8muD9AhVZ6-0KHefVA9QYABABGgJkZw&ohost=www.goo	NO	AD	NO	Ad				NO	Ad
38	Wrist Strain	https://www.googleadservices.com/pagead/aclk?sa=L&ai=DChcSEwih04z8muD9AhVZ6-0KHefVA9QYABAAGgJkZw&ohost=www.goo	NO	Duplicate	NO	Duplicate				NO	Duplicate
39	Wrist Strain	https://www.googleadservices.com/pagead/aclk?sa=L&ai=DChcSEwjh04z8muD9AhVZ6-0KHefVA9QYABACGgJkZw&ohost=www.goo	NO	AD	NO	Ad				NO	Ad
40	Wrist Strain	https://www.googleadservices.com/pagead/aclk?sa=L&ai=DChcSEwjV8Ofbm-D9AhUQhu0KHarhCnAYABABGgJkZw&ohost=www.goo	NO	AD	NO	Ad				NO	Ad
41	Wrist Strain	https://www.googleadservices.com/pagead/aclk?sa=L&ai=DChcSEwjV8Ofbm-D9AhUQhu0KHarhCnAYABAAGgJkZw&ohos	NO	AD	NO	Ad				NO	Ad
42	Wrist Strain	https://www.medicalwesthospital.org/strain-or-sprain-whats-the-difference.php	NO	NO NTWD	NO	No NTWD				NO	Not NTWD
43	Wrist Strain	https://nyulangone.org/conditions/wrist-hand-repetitive-use-injuries/diagnosis	NO	NO NTWD	YES		MB?	MOWP doesn't specify		YES	Non traumatic
44	Wrist Strain	https://www.bouldercentre.com/news/wrist-sprain-common-injury	NO	Duplicate	NO	Duplicate				NO	Duplicate
45	Wrist Strain	https://www.guysandstthomas.nhs.uk/health-information/wrist-sprains-or-strains	NO	NO NTWD	YES		MB?	MOWP doesn't specify		YES	Advice
46	Wrist Strain	https://www.pennmedicine.org/for-patients-and-visitors/patient-information/conditions-treated-a-to-z/wrist-pain	NO	Duplicate	NO	Duplicate				NO	Duplicate
47	Wrist Strain	https://www.handsurgerydenver.com/wrist-sprain-vs-wrist-strain-whats-the-difference/	NO	NO NTWD	YES		MB?	MOWP doesn't specify		YES	mentions both trauma and ntwd
48	Wrist Strain	https://professionalcarept.com/have-you-sustained-a-wrist-sprain-or-strain-a-hand-therapist-could-help/	NO	NO NTWD	YES		MB?	MOWP doesn't specify		YES	Information on NT
49	Wrist Strain	https://www.primarycaresportsmedicine.com/wp-content/uploads/2016/12/WRIST-WRIST-SPRAIN.pdf	NO	NO NTWD	YES		MB?	MOWP doesn't specify		NO	Not NTWD

50	Wrist Strain	https://www.wristsupports.co.uk/wrist-supports-for-wrist-strain.html	NO	AD	NO	Ad			NO	Ad
1	Wrist Pain	https://www.nhs.uk/conditions/hand-pain/wrist-pain/	NO	Duplicate	NO	Duplicate			NO	Duplicate
2	Wrist Pain	https://www.mayoclinic.org/diseases-conditions/wrist-pain/symptoms-causes/syc-20366213	NO	Duplicate	NO	Duplicate			NO	Duplicate
3	Wrist Pain	https://www.versusarthritis.org/about-arthritis/conditions/hand-and-wrist-pain/	NO	Duplicate	NO	Duplicate			NO	Duplicate
4	Wrist Pain	https://www.healthline.com/health/wrist-pain	NO	Duplicate	NO	Duplicate			NO	Duplicate
5	Wrist Pain	https://my.clevelandclinic.org/health/symptoms/17667-wrist-pain	NO	Duplicate	NO	Duplicate			NO	Duplicate
6	Wrist Pain	https://www.medicalnewstoday.com/articles/312070	NO	Duplicate	NO	Duplicate			NO	Duplicate
7	Wrist Pain	https://www.pennmedicine.org/for-patients-and-visitors/patient-information/conditions-treated-a-to-z/wrist-pain	NO	Duplicate	NO	Duplicate			NO	Duplicate
8	Wrist Pain	https://www.voltarol.co.uk/pain-treatments/wrist-pain/	NO	AD	YES	General wrist Info	MB?	Funded ad but wrist pain i	YES	Pain info
9	Wrist Pain	https://www.webmd.com/pain-management/guide/hand-pain-causes	NO	Duplicate	NO	Duplicate			NO	Duplicate
10	Wrist Pain	https://www.arthritis.org/health-wellness/about-arthritis/where-it-hurts/when-hand-or-wrist-pain-may-mean-arthritis	NO	Duplicate	NO	Duplicate			NO	Duplicate
11	Wrist Pain	https://www.googleadservices.com/pagead/aclk?sa=L&ai=DChcSEwjuh6PcnOD9AhVP7e0KHd6HAcoYABAAGgJkZw&ohost=www.go	NO	AD	NO	Ad			NO	Ad
12	Wrist Pain	https://www.googleadservices.com/pagead/aclk?sa=L&ai=DChcSEwjuh6PcnOD9AhVP7e0KHd6HAcoYABABGgJkZw&ohost=www.go	NO	AD	NO	Ad			NO	Ad
13	Wrist Pain	https://www.googleadservices.com/pagead/aclk?sa=L&ai=DChcSEwjuh6PcnOD9AhVP7e0KHd6HAcoYABACGgJkZw&ohost=www.go	NO	AD	NO	Ad			NO	Ad
14	Wrist Pain	https://www.nhsinform.scot/illnesses-and-conditions/muscle-bone-and-joints/self-management-advice/wrist-hand-and-finger-problems	NO	Duplicate	NO	Duplicate			NO	Duplicate
15	Wrist Pain	https://www.verywellhealth.com/wrist-pain-causes-symptoms-and-treatments-2549458	NO	Duplicate	NO	Duplicate			NO	Duplicate
16	Wrist Pain	https://www.aurorahealthcare.org/services/orthopedics/conditions/wrist-pain	YES	Information about ganglion cist	YES				YES	I
17	Wrist Pain	https://www.assh.org/handcare/blog/5-causes-of-wrist-pain	NO	Duplicate	NO	Duplicate			NO	Duplicate
18	Wrist Pain	https://orthoinfo.aaos.org/en/diseases--conditions/carpal-tunnel-syndrome/	NO	NO NTWD	NO	No NTWD			NO	Not NTWD
19	Wrist Pain	https://southernpainclinic.com/blog/wrist-pain-causes-how-to-treat-it/?utm_source=rss&utm_medium=rss&utm_campaign=wrist-pain-c	NO	Duplicate	NO	Duplicate			NO	Duplicate
20	Wrist Pain	https://www.nhsborders.scot.nhs.uk/patients-and-visitors/our-services/allied-health-professionals/physiotherapy/physiotherapy-msk-se	NO	NO NTWD	YES	General pain	MB?	MOWP doesn't specify	NO	Not NTWD
21	Wrist Pain	https://en.wikipedia.org/wiki/Wrist_pain	NO	Journal	YES		MB?	WIKI?	YES	General wrist info
22	Wrist Pain	https://ard.bmj.com/content/58/11/665	NO	Journal	NO	Journal			NO	Journal
23	Wrist Pain	https://www.googleadservices.com/pagead/aclk?sa=L&ai=DChcSEwi-9sLJneD9AhU4gFAGHaBtDCYYABAAGgJkZw&ohost=www.goo	NO	AD	NO	Ad			NO	Ad
24	Wrist Pain	https://www.googleadservices.com/pagead/aclk?sa=L&ai=DChcSEwi-9sLJneD9AhU4gFAGHaBtDCYYABACGgJkZw&ohost=www.goo	NO	AD	NO	Ad			NO	Ad
25	Wrist Pain	https://www.googleadservices.com/pagead/aclk?sa=L&ai=DChcSEwi-9sLJneD9AhU4gFAGHaBtDCYYABABGgJkZw&ohost=www.goo	NO	NO NTWD	NO	No NTWD			NO	Not NTWD
26	Wrist Pain	https://www.assh.org/handcare/condition/ulnar-wrist-pain	NO	Duplicate	NO	Duplicate			NO	Duplicate
27	Wrist Pain	https://www2.hse.ie/conditions/hand-pain/wrist-pain/	NO	Duplicate	NO	Duplicate			NO	Duplicate
28	Wrist Pain	https://www.ncbi.nlm.nih.gov/pmc/articles/PMC1752796/	NO	Journal	NO	Journal			NO	Journal
29	Wrist Pain	https://www.nuffieldhealth.com/symptoms/hand-wrist-pain	NO	AD	YES		MB?	Wrist info	YES	Wrist info
30	Wrist Pain	https://www.mskdorset.nhs.uk/hand-and-wrist-pain/	NO	Duplicate	NO	Duplicate			NO	Duplicate
31	Wrist Pain	https://www.merseycare.nhs.uk/hand-and-wrist-pain	NO	NO NTWD	YES		MB?	MOWP doesn't specify	YES	Wrist info
32	Wrist Pain	https://nyulangone.org/conditions/wrist-hand-repetitive-use-injuries/diagnosis	NO	Duplicate	NO	Duplicate			NO	Duplicate
33	Wrist Pain	https://www.beaumont.org/health-wellness/blogs/hand-and-wrist-pain-what-causes-it-what-can-you-do-about-it	NO	NO NTWD	YES		MB?	No NTWD?	NO	Not NTWD
34	Wrist Pain	https://cich.nhs.uk/services/musculoskeletal-physiotherapy/wrist-and-hand-pain	NO	Video content avaleable NO NTWD umbrella term mentioned	NO	No NTWD	MB?	Confirm reason	NO	Video
35	Wrist Pain	https://www.njpaindoc.com/blog/7-causes-of-wrist-pain-you-should-never-ignore	YES	Ganglion Cist info avaleable	YES				YES	I
36	Wrist Pain	https://www.googleadservices.com/pagead/aclk?sa=L&ai=DChcSEwjl_5llnuD9AhXT7e0KHXNeCHoYABACGgJkZw&ohost=www.goog	NO	AD	NO	Ad			NO	Ad
37	Wrist Pain	https://www.googleadservices.com/pagead/aclk?sa=L&ai=DChcSEwjl_5llnuD9AhXT7e0KHXNeCHoYABAAGgJkZw&ohost=www.goog	NO	AD	NO	Ad			NO	Ad
38	Wrist Pain	https://www.googleadservices.com/pagead/aclk?sa=L&ai=DChcSEwjl_5llnuD9AhXT7e0KHXNeCHoYABABGgJkZw&ohost=www.goog	NO	AD	NO	Ad			NO	Ad
39	Wrist Pain	https://castleortho.co.uk/hand-wrist/wrist-pain/	YES	Included Cyst info interchangeable	YES				YES	I
40	Wrist Pain	How to Fix Wrist Pain for Good - YouTube	NO	Youtube	NO	Video			NO	Video
41	Wrist Pain	https://originhealthgroup.com/pain_injuries/hand-wrist-pain/	NO	NO NTWD	NO	No NTWD			NO	Not NTWD
42	Wrist Pain	https://www.sportsinjuryclinic.net/sport-injuries/wrist-pain-by-location	YES	Included Cyst info interchangeable	YES				YES	I
43	Wrist Pain	https://healthcare.utah.edu/orthopaedics/specialties/hand-pain/when-to-see-a-doctor.php	NO	NO NTWD	YES	General wrist pain	MB?	General wrist pain info?	YES	General wrist pain info
44	Wrist Pain	https://gpnotebook.com/en-gb/simplepage.cfm?ID=624230416	NO	AD?	YES		MB?	Info 1st page?	YES	Wrist pain
45	Wrist Pain	https://www.elastoplast.co.uk/did-you-know/sports-and-activity/wrist-pain-injuries	NO	Duplicate	NO	Duplicate			NO	Duplicate
46	Wrist Pain	https://www.dignityhealth.org/conditions-and-treatments/orthopedics/common-wrist-injuries-and-conditions/wrist-pain	NO	NO NTWD	YES	General wrist pain	MB?	General wrist pain info?	YES	General wrist plan info but mentions TWD
47	Wrist Pain	https://www.newcastle-hospitals.nhs.uk/services/newcastle-occupational-health-service/physiotherapy/wrist-pain/	NO	NO NTWD	YES	General wrist pain	MB?	General wrist pain info?	YES	Wrist pain info
48	Wrist Pain	https://www.nnuh.nhs.uk/publication/download/wrist-and-hand-pain-during-and-after-pregnancy-7/	NO	Condition specific	YES	General wrist pain	MB?	Condition specific?	NO	Specific Demographic
49	Wrist Pain	https://www.googleadservices.com/pagead/aclk?sa=L&ai=DChcSEwiEIKXQnuD9AhUMhu0KHfNDDu0YABABGgJkZw&ohost=www.go	NO	Duplicate	NO	Duplicate			NO	Duplicate
50	Wrist Pain	https://www.googleadservices.com/pagead/aclk?sa=L&ai=DChcSEwiEIKXQnuD9AhUMhu0KHfNDDu0YABACGgJkZw&ohost=www.go	NO	Duplicate	NO	Duplicate			NO	Duplicate
1	Wrist Injury	https://www.nhs.uk/conditions/hand-pain/wrist-pain/	YES	Included Ganglion cyst	YES				YES	I
2	Wrist Injury	https://medlineplus.gov/wristinjuriesanddisorders.html	YES	Included Ganglion Cyst	YES				YES	I
3	Wrist Injury	https://www.mayoclinic.org/diseases-conditions/wrist-pain/symptoms-causes/syc-20366213	YES	Included Ganglion Cyst	YES				YES	I
4	Wrist Injury	https://www.bidneedham.org/departments/orthopaedics/hand-program/most-common-hand-wrist-injuries	NO	Duplicate	NO	Duplicate			NO	Duplicate
5	Wrist Injury	https://www.webmd.com/fitness-exercise/wrist-sprain	NO	NO NTWD	NO	No NTWD			NO	Not NTWD
6	Wrist Injury	https://orthoinfo.aaos.org/en/diseases--conditions/wrist-sprains/	NO	Duplicate	NO	Duplicate			NO	Duplicate
7	Wrist Injury	https://instituteforathleticmedicine.com/specialties/hand-and-wrist/common-hand-and-wrist-injuries/	NO	NO NTWD	YES		MB?	Interchangeable condition	NO	Not NTWD
8	Wrist Injury	https://www.assh.org/handcare/condition/sprained-wrist	NO	NO NTWD	YES		MB?	MOWP doesnt specify	NO	Not NTWD
9	Wrist Injury	https://www.nbt.nhs.uk/our-services/a-z-services/emergency-zone/ed-miu-patient-information/how-treat-your-injured-wrist	NO	NO NTWD	YES		NO	Acute injury	NO	Not NTWD
10	Wrist Injury	https://www.googleadservices.com/pagead/aclk?sa=L&ai=DChcSEwjxup2bmuD9AhUEsO0KHZEnCiQYABAAGgJkZw&ohost=www.go	NO	Duplicate	NO	Duplicate			NO	Duplicate
11	Wrist Injury	https://www.henryford.com/blog/2020/11/wrist-injuries-and-when-to-get-help	YES	Included Cyst info interchangeable	YES				YES	I
12	Wrist Injury	https://www.nhsinform.scot/illnesses-and-conditions/muscle-bone-and-joints/self-management-advice/wrist-hand-and-finger-problems	NO	Duplicate	NO	Duplicate			NO	Duplicate
13	Wrist Injury	https://myhealth.alberta.ca/Health/aftercareinformation/pages/conditions.aspx?hwid=uf7611	NO	Duplicate	NO	Duplicate			NO	Duplicate
14	Wrist Injury	https://www.sports-health.com/sports-injuries/hand-and-wrist-injuries/my-wrist-broken-or-sprained	NO	NO NTWD	YES		MB?	Interchengeable condition	NO	Not NTWD
15	Wrist Injury	https://www.dignityhealth.org/conditions-and-treatments/orthopedics/common-wrist-injuries-and-conditions	YES	Included Cyst info interchangeable	YES				YES	I
16	Wrist Injury	https://www.physio-pedia.com/Hand_and_Wrist_Sports_Injuries	NO	NO NTWD	YES		MB?	Condition Specific	NO	Not NTWD
17	Wrist Injury	https://nyulangone.org/conditions/wrist-hand-repetitive-use-injuries/diagnosis	NO	Duplicate	NO	Duplicate			NO	Duplicate
18	Wrist Injury	https://www.ncbi.nlm.nih.gov/books/NBK551514/	NO	Journal	NO	Journal			NO	Journal
19	Wrist Injury	https://my.clevelandclinic.org/health/diseases/24558-sprained-wrist	NO	Duplicate	NO	Duplicate			NO	Duplicate
20	Wrist Injury	https://www.healthline.com/health/wrist-pain	NO	Duplicate	NO	Duplicate			NO	Duplicate
21	Wrist Injury	https://www.nhsinform.scot/illnesses-and-conditions/muscle-bone-and-joints/fractures/wrist-fracture	NO	NO NTWD	NO	No NTWD			NO	Not NTWD
22	Wrist Injury	https://www.nationwidechildrens.org/specialties/sports-medicine/sports-medicine-articles/wrist-sprains	NO	NO NTWD	YES		MB?	MOWP doesnt specify	NO	Not NTWD
23	Wrist Injury	https://elht.nhs.uk/application/files/2115/8860/9033/Wrist_Injury_leaflet_V4.pdf	NO	NO NTWD	YES		MB?	Population specific & injur	YES	General wrist info
24	Wrist Injury	https://www.emedicinehealth.com/wrist_injury/article_em.htm	NO	NO NTWD	YES		MB?	Interchengeable condition	YES	Not Trauma + General wrist info

25	Wrist injury	https://www.hey.nhs.uk/patient-leaflet/soft-tissue-injury-wrist-hand/	NO	NO NTWD	YES	Injury not traumtic	MB?	Injury specific?	YES	Not trauma
26	Wrist injury	https://www.bmj.com/content/361/bmj.k2574	NO	Journal	NO	Jounral			NO	Journal
27	Wrist injury	https://www.massgeneralbrigham.org/en/patient-care/services-and-specialties/sports-medicine/conditions/hand-arm/wrist-sprain	NO	NO NTWD	YES		MB?	MOWP doesnt specify	NO	Not NTWD
28	Wrist injury	https://www.pennmedicine.org/for-patients-and-visitors/patient-information/conditions-treated-a-to-z/wrist-pain	NO	Duplicate	NO	Duplicate			NO	Duplicate
29	Wrist injury	https://www.jmw.co.uk/services-for-you/personal-injury/compensation-calculator/arm-injury/wrist-injury	NO	AD	NO	Ad			NO	Ad
30	Wrist injury	https://www.bouldercentre.com/news/wrist-sprain-common-injury	NO	NO NTWD	YES		MB?	MOWP doesnt specify	NO	Duplicate
31	Wrist injury	https://www.googleadservices.com/pagead/aclk?sa=L&ai=DChcSEwibvdDemuD9AhWJtu0KHWVxAYYYABACGgJkZw&ohost=www.g	NO	AD	NO	Ad			NO	Ad
32	Wrist injury	https://www.googleadservices.com/pagead/aclk?sa=L&ai=DChcSEwibvdDemuD9AhWJtu0KHWVxAYYYABABGgJkZw&ohost=www.g	NO	AD	NO	Ad			NO	Ad
33	Wrist injury	https://www.googleadservices.com/pagead/aclk?sa=L&ai=DChcSEwibvdDemuD9AhWJtu0KHWVxAYYYABAAGgJkZw&ohost=www.g	NO	Duplicate	NO	Duplicate			NO	Duplicate
34	Wrist injury	https://www.chelwest.nhs.uk/your-visit/patient-leaflets/hand-therapy/managing-with-a-hand-or-wrist-injury	NO	NO NTWD	NO	No NTWD			NO	Not NTWD
35	Wrist injury	https://www.elastoplast.co.uk/did-you-know/sports-and-activity/wrist-pain-injuries	NO	AD	YES		YES	Wrist info (GANGLION CI	YES	I
36	Wrist injury	https://www.drugs.com/health-guide/wrist-sprain.html	YES	Included and ganglion cyst included	YES				YES	I
37	Wrist injury	https://www.verywellhealth.com/wrist-pain-causes-symptoms-and-treatments-2549458	NO	Duplicate	NO	Duplicate			NO	Duplicate
38	Wrist injury	https://handandwristinstitute.com/whats-better-for-a-wrist-sprain-ice-or-heat/	NO	NO NTWD	YES	Sprains	MB?	MOWP doesnt specify	YES	Chronic strain info
39	Wrist injury	https://www.hss.edu/condition-list_scaphoid-injuries-wrist-pain.asp	NO	NO NTWD	NO	No NTWD			NO	Not NTWD
40	Wrist injury	http://www.mtw.nhs.uk/wp-content/uploads/2015/11/Leaflet-wrist-sprain.pdf	NO	Duplicate	NO	Duplicate			NO	Duplicate
41	Wrist injury	https://www.gloshospitals.nhs.uk/documents/8047/Soft_tissue_injuries_affecting_the_wrist_and_hand_GHPI0878_04_19_p0l6Hy7.pdf	NO	NO NTWD	YES		MB?	Soft tissue injury?	YES	General soft tissue injury info
42	Wrist injury	http://www.buckinghamshirehandsurgeon.co.uk/conditions-treated/ligament-and-soft-tissue-wrist-injury/	NO	NO NTWD	YES		YES	TFCC information present	YES	I
43	Wrist injury	https://yogainternational.com/article/view/healing-and-preventing-wrist-injuries	NO	NO NTWD	YES	Generic Wrist	MB?	Sport specific	YES	Generic wrist info but aimed at yoga
44	Wrist injury	https://www.ruh.nhs.uk/patients/patient_information/ORT_057_Advice_after_a_wrist_sprain.pdf	NO	NO NTWD	YES	Sprain	MB?	MOWP doesnt specify	YES	Sprain
45	Wrist injury	https://www.sportsmedtoday.com/ulnarsided-wrist-injuries-va-61.htm	YES	Included	YES				YES	I
46	Wrist injury	https://www.choa.org/medical-services/orthopedics/wrist-hand-injuries	NO	NO NTWD	YES		MB?	Peadiatric	NO	Specific Demographic
47	Wrist injury	https://www.northdevonhealth.nhs.uk/wp-content/uploads/2021/05/Protocol-for-the-Management-of-wrist-injuries-in-MIUs-V2.0.pdf	NO	NO NTWD	NO	Professional use			NO	Not NTWD
48	Wrist injury	https://www.usta.com/en/home/improve/tennis-health---fitness/national/mount-sinai-health-tip--it-s-in-the-wrist.html	NO	NO NTWD	YES	Wrist info	MB?	Sport specific	NO	Specific Demographic
49	Wrist injury	https://www.medicalnewstoday.com/articles/312070	NO	Duplicate	NO	Duplicate			NO	Duplicate
50	Wrist injury	https://www.woodlandssportsmedicine.com/blog/5-common-hand-and-wrist-injuries-among-athletes	NO	NO NTWD	YES	NTWD	MB?	Interchangeable condition	YES	Mentions both trauma and non-trauma
1	Why does my	https://www.mayoclinic.org/diseases-conditions/wrist-pain/symptoms-causes/syc-20366213#:~:text=Wrist%20pain%20is%20often%20	YES	Included Ganglion Cyst	NO	Duplicate	MB?	Duplicate?	YES	Can't find a dupe
2	Why does my	https://www.nhs.uk/conditions/hand-pain/wrist-pain/	NO	Duplicate	NO	Duplicate			NO	Duplicate
3	Why does my	https://www.medicalnewstoday.com/articles/312070	YES	Included Ganglion Cyst	YES				YES	I
4	Why does my	https://my.clevelandclinic.org/health/symptoms/17667-wrist-pain	NO	Duplicate	NO	Duplictae			NO	Duplicate
5	Why does my	https://www.pennmedicine.org/for-patients-and-visitors/patient-information/conditions-treated-a-to-z/wrist-pain	NO	Duplicate	YES		MB?	Dupliactes mismatch?	NO	Duplicate
6	Why does my	https://www.verywellhealth.com/wrist-pain-causes-symptoms-and-treatments-2549458	YES	Included	YES		MB?	Dupliactes mismatch?	YES	1st Duplicate
7	Why does my	https://www.webmd.com/pain-management/guide/hand-pain-causes	NO	Duplicate	YES	General wrist	MB?	General wrist info?	YES	Wrist info
8	Why does my	https://www.healthline.com/health/wrist-pain	NO	Duplicate	YES	General Wrist pain	MB?	Dupliactes mismatch? + g	YES	1st Duplicate
9	Why does my	https://www.versusarthritis.org/about-arthritis/conditions/hand-and-wrist-pain/	NO	Duplicate	YES	General wrist	MB?	Duplicate mismatch	NO	Duplicate
10	Why does my	https://www.njpaindoc.com/blog/7-causes-of-wrist-pain-you-should-never-ignore	NO	Duplicate	NO	Duplicate	MB?	Duplicate mismatch ?	NO	Duplicate
11	Why does my	https://www.blog.ohmyarthritis.com/my-wrist-hurts-common-causes-of-wrist-pain/	YES	Included Ganglion Cyst	NO	Not accessible - AVG stopped	Yes		YES	I
12	Why does my	https://www.beaumont.org/health-wellness/blogs/hand-and-wrist-pain-what-causes-it-what-can-you-do-about-it	NO	Duplicate	NO	Duplicate			NO	Duplicate
13	Why does my	https://www.nhsinform.scot/illnesses-and-conditions/muscle-bone-and-joints/self-management-advice/wrist-hand-and-finger-problems	NO	Duplicate	YES		MB?	Duplicate mismatch? + ge	YES	1st Duplicate + general wrist info
14	Why does my	https://southernpainclinic.com/blog/wrist-pain-causes-how-to-treat-it/?utm_source=rss&utm_medium=rss&utm_campaign=wrist-pain-c	NO	Duplicate	YES		YES	NTWD + check Duplicate	YES	I
15	Why does my	https://www.arthritis.org/health-wellness/about-arthritis/where-it-hurts/when-hand-or-wrist-pain-may-mean-arthritis	NO	NO NTWD	NO	No NTWD			NO	Not NTWD
16	Why does my	https://hordercentre.co.uk/news/hand-therapy-qa-what-is-causing-my-wrist-pain-at-the-gym/	NO	Duplicate	NO	Duplicate			NO	Duplicate
17	Why does my	https://www.assh.org/handcare/blog/5-causes-of-wrist-pain	NO	Duplicate	YES	NTWD	YES	NTWD + check Duplicate	YES	I
18	Why does my	https://www.assh.org/handcare/condition/ulnar-wrist-pain	YES	Included NTWD	YES	NTWD	MB?	YES - Same website (dupl	YES	1st Duplicate
19	Why does my	https://www.urmc.rochester.edu/orthopaedics/hand-wrist/conditions.cfm	NO	Duplicate	YES		MB?	Duplicate mismatch + NT	YES	1st Duplicate + info
20	Why does my	https://www.googleadservices.com/pagead/aclk?sa=L&ai=DChcSEwik4rineD9AhWD6-0KHbDxA4AYABAAGgJkZw&ohost=www.goog	NO	AD	NO	Duplicate	MB?	Ad or Duplicate?	NO	Ad
21	Why does my	https://www.googleadservices.com/pagead/aclk?sa=L&ai=DChcSEwik4rineD9AhWD6-0KHbDxA4AYABACGgJkZw&ohost=www.goog	NO	Duplicate	YES	NTWD	MB?	Duplicate mismatch + NT	NO	Ad
22	Why does my	https://www.geisinger.org/health-and-wellness/wellness-articles/2018/10/04/20/06/dont-ignore-your-hand-and-wrist-pain-here-are-the-t	YES	Included Cyst info interchangeable	YES				YES	I
23	Why does my	https://www.ivyrehab.com/news/common-causes-for-sharp-pain-in-wrists/	NO	NO NTWD	YES	General wrist pain	MB?	General wrist info	YES	General wrist info
24	Why does my	https://www.bordertherapy.com/why-does-my-wrist-hurt-when-i-bend-it-or-put-pressure-on-it/	NO	NO NTWD	YES	Sprain	MB?	MOWP doesnt specify (wr	YES	Sprain info
25	Why does my	https://orthoinfo.aaos.org/en/diseases--conditions/wrist-sprains/	NO	Duplicate	NO	Duplicate			NO	Duplicate
26	Why does my	https://nyulangone.org/conditions/wrist-hand-repetitive-use-injuries/diagnosis	NO	Duplicate	NO	Duplicate			NO	Duplicate
27	Why does my	https://healthcare.utah.edu/orthopaedics/specialties/hand-pain/when-to-see-a-doctor.php	NO	Doulicate	NO	Duplicate			NO	Duplicate
28	Why does my	https://www.templehealth.org/about/blog/is-your-wrist-pain-carpal-tunnel-syndrome	NO	NO NTWD	NO	No NTWD			NO	Not NTWD
29	Why does my	https://myhealth.alberta.ca/Health/aftercareinformation/pages/conditions.aspx?hwid=uf7611	NO	Duplicate	NO	Duplicate			NO	Duplicate
30	Why does my	https://handinjuries.com.au/index.php/blog1/entry/why-does-my-wrist-hurt-and-cause-me-pain	YES	Incuded cyst info	YES				YES	I
31	Why does my	https://www2.hse.ie/conditions/hand-pain/wrist-pain/	NO	Duplicate	YES		MB?	YES - Same website (dupl	YES	1st Duplicate
32	Why does my	https://www.googleadservices.com/pagead/aclk?sa=L&ai=DChcSEwik4rineD9AhWD6-0KHbDxA4AYABABGgJkZw&ohost=www.goog	NO	AD	NO	Ad			NO	Ad
33	Why does my	https://www.sports-health.com/sports-injuries/hand-and-wrist-injuries/symptoms-wrist-tendonitis	NO	NO NTWD	YES	General wrist and tendonitis	MB?	No NTWD? and same wel	YES	General wrist info
34	Why does my	https://patients.stryker.com/wrist-replacement/understanding-wrist-pain	NO	NO NTWD	NO	No NTWD			NO	Not NTWD
35	Why does my	https://adventpt.com/top-of-wrist-pain-it-might-not-be-carpal-tunnel-syndrome/	NO	NO NTWD	YES	Tendonitis/general wrist	MB?	Confirm if NTWD definitio	YES	Tendonitis + general wrist info
36	Why does my	https://carrolltonortho.com/news-events/posts/wrist/8-ways-to-easily-treat-and-prevent-wrist-pain/	NO	NO NTWD	YES	General wrist pain	MB?	General wrist pain info? N	YES	General wrist info
37	Why does my	https://newyorkhandandnerve.com/services/hand-upper-extremity/causes-hand-wrist-pain/	NO	NO NTWD	YES	NTWD	YES	Tendonitous info included	YES	I

38	Why does my (	https://www.bidneedham.org/departments/orthopaedics/hand-program/most-common-hand-wrist-injuries	NO	Duplicate	YES	NTWD / Sprains	MB?	Duplicate mismatch? + ch	NO	Duplicate
39	Why does my (	https://familydoctor.org/condition/de-quervains-tenosynovitis/	NO	NO NWTD	YES	NTWD	YES	Agreed on NTWD content	YES	I
40	Why does my (	https://www.henryford.com/blog/2017/10/carpal-tunnel-vs-tendonitis-identifying-symptoms	YES	Included TFCC	YES	NTWD			YES	I
41	Why does my (	https://www.healthgrades.com/right-care/bones-joints-and-muscles/7-common-causes-of-wrist-pain	NO	Not acceseable	NO	Not accessible			NO	Not publicaly Accessible
42	Why does my (	https://medicine.umich.edu/dept/orthopaedic-surgery/patient-care-services-hand-upper-extremity/post-traumatic-wrist-arthritis	NO	NO NTWD	YES	Arthritis	MB?	Risk of OA Post traumatic	YES	OA of wrist is ntwd
43	Why does my (	https://www.googleadservices.com/pagead/aclk?sa=L&ai=DChcSEwiC2ZKmuD9AhUB3u0KHeXdCf8YABACGgJkZw&ohost=www.goo	NO	Seach Engine	NO	Search			NO	Search Engine
44	Why does my (	https://www.googleadservices.com/pagead/aclk?sa=L&ai=DChcSEwiC2ZKmuD9AhUB3u0KHeXdCf8YABABGgJkZw&ohost=www.goo	NO	AD	NO	Ad			NO	Ad
45	Why does my (	https://www.googleadservices.com/pagead/aclk?sa=L&ai=DChcSEwiC2ZKmuD9AhUB3u0KHeXdCf8YABAAGgJkZw&ohost=www.goo	NO	Duplicate	NO	Duplicate			NO	Duplicate
46	Why does my (	https://sportdoctorlondon.com/wrist-pain-and-popping/	YES	Included TFCC	YES				YES	I
47	Why does my (	https://twinboro.com/why-does-my-wrist-hurt	NO	NO NTWD	YES	Generic wrist pain	MB?	General wrist info no spec	YES	General wrist info
48	Why does my (	https://yorkvillephysiotherapy.com/why-does-the-pinky-side-of-my-wrist-hurt/	YES	Included TFCC	YES				YES	I
49	Why does my (	https://www.buoyhealth.com/learn/wrist-pain	NO	NO NTWD	YES	NTWD	MB?	Duplicate mismatch? Bou	YES	Not duplicate, wrist info
50	Why does my (	https://www.wikihow.com/Relieve-Wrist-Pain-from-Lifting	NO	NO NTWD	YES	Wrist Pain	MB?	Function specific? not cla	YES	general wrist info

Number of Incl	85
Duplicate	101
Ad	38
Not NTWD	50
Journal	6
Video	5
Music	1
Not publically a	3
Interactive	1
Search Engine	5
Specific Demo	5
total	215

Additional source characteristics

				Additional factors. Click home keybind to return to start								
Website URL	Who is the website funded by?	What is the country of origin for the website?	What management technique/s is/are recommended	Does it recommend a frequency for the intervention?	Does it provide a length of time to adhere to the intervention?	Does it provide a length of time for recovery?	Does it mention global health factors?	How many references are mentioned?	Date of creation	Date of last update	Score of additional factors	Comments
https://eastendot.com/what-can-i-do-for-weak-wrists-occupational-therapy/	Private Service	USA	Exercise; Splinting;	No	No	No	No	1	Not Found	2023	1	
https://seriouslystrongtraining.com/6-tips-to-strengthen-weak-painful-wrists/	Private Service	USA	Exercise	No	No	No	No	0	Not Found	10/4/2023	0	
https://www.medicalnewstoday.com/articles/hand-weakness	Medical info outlet	England	Exercise; Medication; Surgery; Splinting; CT; Injection	Yes	No	Yes	Yes	3	22/12/2020	22/12/2020	4	pregnancy, dietry(protein), diabetes, alcohol,
https://www.wristsupports.co.uk/blog/what-is-general-wrist-weakness.html	Product	England	Exercise; HT; CT;	No	No	No	No	0	23/8/2017	23/8/2017	0	
https://my.clevelandclinic.org/health/symptoms/17667-wrist-pain	Private Service	USA	Exercise; Injection;	No	No	No	No	4	11/3/2017	11/3/2017	1	
https://tommorrison.uk/blog/fix-your-weak-wrists	Private Blog	Northern Ireland	Exercise	No	No	No	No	0	Not Found	Not Found	0	
https://www.healthline.com/health/how-to-strengthen-wrists	Medical info outlet	USA	Exercise; Splinting;	No	No	No	No	9	Not Found	8/7/2019	1	
https://www.buoyhealth.com/learn/hand-weakness	Clinical Website	USA	Splinting; Medication; Surgery; Exercise	No	No	No	Yes	5	Not Found	14/10/2021	2	cardiovascular disease, body mass
https://orthoinfo.aaos.org/en/diseases--conditions/ulnar-tunnel-syndrome-of-the-wrist/	Clinical Website	USA	Surgery; Exercise;	No	No	No	No	1	28/2/2022	28/2/2022	1	
https://experiencelife.lifetime.life/article/fitness-fix-help-for-weak-wrists/	Wellbeing Website	USA	Exercise	No	No	No	No	0	30/6/2014	30/6/2014	0	
https://www.beachbodyondemand.com/blog/modified-exercises-for-weak-wrists	Private Blog	USA	Exercise	No	No	No	No	0	31/1/2023	31/1/2023	0	
https://www.verywellhealth.com/wrist-strengthening-exercises-2696622	Wellbeing Website	USA	Exercise	No	No	No	No	1	11/11/2022	11/11/2022	1	
https://medi-dyne.com/pages/injury-treatment-wrist-pain	Product	USA	Splinting; Exercise;	No	No	No	No	0	Not Found	Not Found	0	
https://www.versusarthritis.org/about-arthritis/conditions/osteoarthritis-of-the-hand-and-wrist/	Charity	England	Lifestyle;	No	No	No	No	0	Not Found	Not Found	0	
https://sportsmedicine.mayoclinic.org/condition/wrist-sprains/	Clinical Website	USA	Rest; Splinting; CT;	No	No	No	No	0	Not Found	Not Found	0	
http://www.mtw.nhs.uk/wp-content/uploads/2015/11/Leaflet-wrist-sprain.pdf	NHS	England	Medication; Rest;	Yes	No	Yes	No	0	Not Found	Not Found	2	
https://www.mskdiagnostics.co.uk/patients/resources/hand-and-wrist/wrist-sprain/	Private Service	England	CT; HT;	No	No	Yes	No	0	Not Found	Not Found	1	
https://www.bonsecours.com/health-care-services/orthopedics-sports-medicine/hand-wrist/conditions/hand-a	Private Service	USA	Rest; HT; CT;	No	No	No	No	0	Not Found	19/4/2023	0	
https://www.nhs.uk/conditions/sprains-and-strains/	NHS	England	PT; Medication;	Yes	No	Yes	No	0	10/2/2021	10/2/2021	2	
https://www.sportsinjuryclinic.net/sport-injuries/wrist-pain/acute-wrist-injuries/wrist-strain	Private Service	ENgland	Medication; RICE;	No	Yes	No	No	0	1/2/2023	1/2/2023	1	
https://www.physio.co.uk/what-we-treat/musculoskeletal/conditions/wrist/wrist-strain.php	Private Service	England	HT; PT; Splinting;	No	No	No	No	0	Not Found	Not Found	0	
https://www.sports-health.com/sports-injuries/hand-and-wrist-injuries/wrist-tendonitis-vs-sprain	Clinical Website	USA	HT; Medication;	No	Yes	No	No	0	10/2/23	9/2/2023	1	
https://www.bsuh.nhs.uk/wp-content/uploads/sites/5/2016/09/Wrist-sprain.pdf	NHS	England	Medication; CT;	Yes	Yes	Yes	No	0	1/8/2013	1/8/15	3	
https://nyulangone.org/conditions/wrist-hand-repetitive-use-injuries/diagnosis	Private Service	USA	RICE; HT; Injection;	No	No	No	No	0	Not Found	Not Found	0	
https://www.guysandstthomas.nhs.uk/health-information/wrist-sprains-or-strains	NHS	England	CT; Rest; Exercise;	No	No	No	No	0	1/11/2019	1/11/22	0	
https://www.handsurgerydenver.com/wrist-sprain-vs-wrist-strain-whats-the-difference/	Private Service	USA	Surgery;	No	No	No	No	0	Not Found	Not Found	0	
https://professionalcarept.com/have-you-sustained-a-wrist-sprain-or-strain-a-hand-therapist-could-help/	Private Service	USA	MT; PT	No	No	No	No	1	10/12/2021	Not Found	1	
https://www.voltarol.co.uk/pain-treatments/wrist-pain/	Product	England	Rest; CT;	No	No	No	Yes	1	Not Found	Not Found	2	overweight,
https://www.aurorahealthcare.org/services/orthopedics/conditions/wrist-pain	Private Service	USA	Rest; CT; Splinting;	No	No	No	No	0	Not Found	Not Found	0	
https://en.wikipedia.org/wiki/Wrist_pain	Online encyclopedia	USA	RICE; Activity	No	No	Yes	No	20	Not Found	12/3/2023	2	
https://www.nuffieldhealth.com/symptoms/hand-wrist-pain	Private Service	England	RICE; CT; Medication	No	No	No	No	0	Not Found	Not Found	0	
https://www.merseycare.nhs.uk/hand-and-wrist-pain	NHS	England	CT; Medication;	No	No	No	No	0	Not Found	Not Found	0	
https://www.njpaindoc.com/blog/7-causes-of-wrist-pain-you-should-never-ignore	Private Service	USA	Splint; Injection;	No	No	No	No	0	Not Found	Not Found	0	
https://castleortho.co.uk/hand-wrist/wrist-pain/	Private Service	ENgland	Medication;	No	No	No	No	0	Not Found	Not Found	0	
https://www.sportsinjuryclinic.net/sport-injuries/wrist-pain-by-location	Private Service	England	Medication; CT;	No	No	No	No	0	Not Found	10/4/2023	0	
https://healthcare.utah.edu/orthopaedics/specialties/hand-pain/when-to-see-a-doctor.php	Private Service	USA	RICE; Medication;	No	No	Yes	No	0	Not Found	Not Found	1	
https://gpnotebook.com/en-gb/simplepage.cfm?ID=624230416	Medical info outlet	England	Rest; Injection;	No	No	No	No	3	Not Found	1/9/2021	1	
https://www.dignityhealth.org/conditions-and-treatments/orthopedics/common-wrist-injuries-and-conditions/	Private Service	USA	Rest; Splinting;	No	No	Yes	No	0	Not Found	Not Found	1	
https://www.newcastle-hospitals.nhs.uk/services/newcastle-occupational-health-service/physiotherapy/wrist-p	NHS	England	Medication; HT; CT;	Yes	Yes	No	No	0	9/2/23	9/2/2023	2	
https://www.nhs.uk/conditions/hand-pain/wrist-pain/	NHS	England	RICE; Medication;	Yes	Yes	No	No	0	Not Found	8/3/2022	2	
https://medlineplus.gov/wristinjuriesanddisorders.html	Medical info outlet	USA	RICE; Surgery;	No	No	No	No	2	6/8/2022	6/8/2022	1	
https://www.mayoclinic.org/diseases-conditions/wrist-pain/symptoms-causes/syc-20366213	Private Service	USA	Rest; CT;	No	No	No	No	15	Not Found	Not Found	1	
https://www.henryford.com/blog/2020/11/wrist-injuries-and-when-to-get-help	Private Service	USA	Splinting	No	No	Yes	No	0	10/11/2020	10/11/2020	1	

https://www.sports-health.com/sports-injuries/hand-and-wrist-injuries/wrist-tendonitis-vs-sprain	Clinical Website	USA	HT; Medication;	No	Yes	No	No	0	10/2/23	9/2/2023	1	
https://www.bsuh.nhs.uk/wp-content/uploads/sites/5/2016/09/Wrist-sprain.pdf	NHS	England	Medication; CT;	Yes	Yes	Yes	No	0	1/8/2013	1/8/15	3	
https://nyulangone.org/conditions/wrist-hand-repetitive-use-injuries/diagnosis	Private Service	USA	RICE; HT; Injection;	No	No	No	No	0	Not Found	Not Found	0	
https://www.guysandstthomas.nhs.uk/health-information/wrist-sprains-or-strains	NHS	England	CT; Rest; Exercise;	No	No	No	No	0	1/11/2019	1/11/22	0	
https://www.handsurgerydenver.com/wrist-sprain-vs-wrist-strain-whats-the-difference/	Private Service	USA	Surgery;	No	No	No	No	0	Not Found	Not Found	0	
https://professionalcarept.com/have-you-sustained-a-wrist-sprain-or-strain-a-hand-therapist-could-help/	Private Service	USA	MT; PT	No	No	No	No	1	10/12/2021	Not Found	1	
https://www.voltarol.co.uk/pain-treatments/wrist-pain/	Product	England	Rest; CT;	No	No	No	Yes	1	Not Found	Not Found	2	overweight,
https://www.aurorahealthcare.org/services/orthopedics/conditions/wrist-pain	Private Service	USA	Rest; CT; Splinting;	No	No	No	No	0	Not Found	Not Found	0	
https://en.wikipedia.org/wiki/Wrist_pain	Online encyclopedia	USA	RICE; Activity	No	No	Yes	No	20	Not Found	12/3/2023	2	
https://www.nuffieldhealth.com/symptoms/hand-wrist-pain	Private Service	England	RICE; CT; Medication	No	No	No	No	0	Not Found	Not Found	0	
https://www.merseycare.nhs.uk/hand-and-wrist-pain	NHS	England	CT; Medication;	No	No	No	No	0	Not Found	Not Found	0	
https://www.njpaindoc.com/blog/7-causes-of-wrist-pain-you-should-never-ignore	Private Service	USA	Splint; Injection;	No	No	No	No	0	Not Found	Not Found	0	
https://castleortho.co.uk/hand-wrist/wrist-pain/	Private Service	ENgland	Medication;	No	No	No	No	0	Not Found	Not Found	0	
https://www.sportsinjuryclinic.net/sport-injuries/wrist-pain-by-location	Private Service	England	Medication; CT;	No	No	No	No	0	Not Found	10/4/2023	0	
https://healthcare.utah.edu/orthopaedics/specialties/hand-pain/when-to-see-a-doctor.php	Private Service	USA	RICE; Medication;	No	No	Yes	No	0	Not Found	Not Found	1	
https://gpnotebook.com/en-gb/simplepage.cfm?ID=624230416	Medical info outlet	England	Rest; Injection;	No	No	No	No	3	Not Found	1/9/2021	1	
https://www.dignityhealth.org/conditions-and-treatments/orthopedics/common-wrist-injuries-and-conditions/v	Private Service	USA	Rest; Splinting;	No	No	Yes	No	0	Not Found	Not Found	1	
https://www.newcastle-hospitals.nhs.uk/services/newcastle-occupational-health-service/physiotherapy/wrist-p	NHS	England	Medication; HT; CT;	Yes	Yes	No	No	0	9/2/23	9/2/2023	2	
https://www.nhs.uk/conditions/hand-pain/wrist-pain/	NHS	England	RICE; Medication;	Yes	Yes	No	No	0	Not Found	8/3/2022	2	
https://medlineplus.gov/wristinjuriesanddisorders.html	Medical info outlet	USA	RICE; Surgery;	No	No	No	No	2	6/8/2022	6/8/2022	1	
https://www.mayoclinic.org/diseases-conditions/wrist-pain/symptoms-causes/syc-20366213	Private Service	USA	Rest; CT;	No	No	No	No	15	Not Found	Not Found	1	
https://www.henryford.com/blog/2020/11/wrist-injuries-and-when-to-get-help	Private Service	USA	Splinting	No	No	Yes	No	0	10/11/2020	10/11/2020	1	
https://elht.nhs.uk/application/files/2115/8860/9033/Wrist_injury_leaftlet_V4.pdf	NHS	England	Exercise; CT	Yes	No	Yes	No	0	1/4/2020	1/4/2020	2	
https://www.emedicinehealth.com/wrist_injury/article_em.htm	Medical info outlet	USA	Splinting;	No	No	Yes	No	1	1/11/2022	1/11/2022	2	
https://www.hey.nhs.uk/patient-leaflet/soft-tissue-injury-wrist-hand/	NHS	ENgland	RICE; Exercise	Yes	Yes	Yes	Yes	0	19/1/2021	19/1/2022	4	alcohol,
https://www.elastoplast.co.uk/did-you-know/sports-and-activity/wrist-pain-injuries	Product	England	RICE; Splinting	No	No	No	No	0	Not Found	Not Found	0	
https://www.drugs.com/health-guide/wrist-sprain.html	Medical info outlet	USA	RICE; Medication;	No	No	Yes	No	0	23/1/2023	23/1/2023	1	
https://handandwristinstitute.com/whats-better-for-a-wrist-sprain-ice-or-heat/	Private Service	USA	RICE; HT	No	Yes	No	No	0	Not Found	Not Found	1	
https://www.gloshospitals.nhs.uk/documents/8047/Soft_tissue_injuries_affecting_the_wrist_and_hand_GHPI0	NHS	England	RICE; CT; Exercise;	Yes	Yes	Yes	No	0	1/4/2019	1/4/2022	3	
http://www.buckinghamshirehandsurgeon.co.uk/conditions-treated/ligament-and-soft-tissue-wrist-injury/	Private Service	England	Medication;	No	No	Yes	No	0	Not Found	Not Found	1	
https://yogainternational.com/article/view/healing-and-preventing-wrist-injuries	Private Blog	USA	Yoga	No	No	No	No	0	Not Found	Not Found	0	
https://www.ruh.nhs.uk/patients/patient_information/ORT_057_Advice_after_a_wrist_sprain.pdf	NHS	England	Splinting;	No	Yes	Yes	Yes	0	1/2/2020	Not Found	3	smoking

https://www.sportsmedtoday.com/ulnarsided-wrist-injuries-va-51.htm	Medical info outlet	USA	Splinting; Taping;	No	No	No	No	2	Not Found	Not Found	1	
https://www.woodlandssportsmedicine.com/blog/5-common-hand-and-wrist-injuries-among-athletes	Private Service	USA	Rest; CT;	Yes	No	No	No	0	Not Found	Not Found	1	
https://www.mayoclinic.org/diseases-conditions/wrist-pain/symptoms-causes/syc-20366213#:~:text=Wrist%20	Private Service	USA	Medication;	No	No	No	Yes	0	28/10/2022	Not Found	1	pregnancy,
https://www.medicalnewstoday.com/articles/312070	Medical info outlet	USA	Rest; CT;	No	No	No	Yes	7	23/11/2021	Not Found	2	diabetes,
https://www.verywellhealth.com/wrist-pain-causes-symptoms-and-treatments-2549458	Medical info outlet	USA	RICE; Splinting;	No	Yes	No	Yes	0	12/10/2022	Not Found	2	sis, gout,
https://www.webmd.com/pain-management/guide/hand-pain-causes	Medical info outlet	USA	Splinting;	No	No	No	Yes	0	18/12/2022	Not Found	1	diabetes
https://www.healthline.com/health/wrist-pain	Medical info outlet	USA	Medication; HT;	No	No	No	Yes	12	31/1/2022	Not Found	2	diabetes,
https://www.nhsinform.scot/illnesses-and-conditions/muscle-bone-and-joints/self-management-advice/wrist-h	NHS	England	Medication; HT; CT;	Yes	No	Yes	Yes	0	2/2/2023	Not Found	3	body mass,
https://southernpainclinic.com/blog/wrist-pain-causes-how-to-treat-it/?utm_source=rss&utm_medium=rss&utr	Private Service	USA	Medication; RICE;	No	No	No	No	0	16/11/2022	Not Found	0	
https://www.assh.org/handcare/blog/5-causes-of-wrist-pain	Private Service	USA	Surgery; Splinting;	No	No	No	No	0	22/5/2016	Not Found	0	
https://www.assh.org/handcare/condition/ulnar-wrist-pain	Private Service	USA	Surgery; Splinting;	No	No	No	No	0	Not Found	Not Found	0	
https://www.urmc.rochester.edu/orthopaedics/hand-wrist/conditions.cfm	Private Service	USA	Surgery; Splinting;	No	No	No	No	0	Not Found	Not Found	0	
https://www.geisinger.org/health-and-wellness/wellness-articles/2018/10/04/20/06/dont-ignore-your-hand-and	Private Service	USA	Surgery;	No	No	No	No	0	13/6/2022	Not Found	0	
https://www.ivyrehab.com/news/common-causes-for-sharp-pain-in-wrists/	Private Service	USA	Medication;	No	No	No	No	0	20/9/2020	Not Found	0	
https://www.bordertherapy.com/why-does-my-wrist-hurt-when-i-bend-it-or-put-pressure-on-it/	Private Service	USA	MT; Taping; Dry	No	No	No	No	0	Not Found	Not Found	0	
https://handinjuries.com.au/index.php/blog1/entry/why-does-my-wrist-hurt-and-cause-me-pain	Private Blog	AUS	Splinting	No	No	No	No	0	Not Found	Not Found	0	
https://www2.hse.ie/conditions/hand-pain/wrist-pain/	Private Service	Ireland	Rest; Splinting;	No	Yes	No	No	0	8/4/2021	8/4/2024	1	
https://www.sports-health.com/sports-injuries/hand-and-wrist-injuries/symptoms-wrist-tendonitis	Medical info outlet	USA	Splinting;	No	Yes	Yes	Yes	9	4/4/2019	4/4/2019	4	high
https://adventpt.com/top-of-wrist-pain-it-might-not-be-carpal-tunnel-syndrome/	Private Service	USA	PT; Splinting; Taping	No	No	Yes	No	0	Not Found	Not Found	1	
https://carrolltonortho.com/news-events/posts/wrist/8-ways-to-easily-treat-and-prevent-wrist-pain/	Private Service	USA	Exercise; Activity	No	No	No	No	0	3/1/2020	Not Found	0	
https://newyorkhandandnerve.com/services/hand-upper-extremity/causes-hand-wrist-pain/	Private Service	USA	Medication;	No	No	No	No	7	Not Found	Not Found	1	
https://familydoctor.org/condition/de-quervains-tenosynovitis/	Private Service	USA	HT; CT; Medication;	No	Yes	Yes	No	0	6/12/2022	6/12/2022	2	
https://www.henryford.com/blog/2017/10/carpal-tunnel-vs-tendonitis-identifying-symptoms	Private Blog	USA	Medication; CT;	No	No	No	No	0	30/10/2017	30/10/2022	0	
https://medicine.umich.edu/dept/orthopaedic-surgery/patient-care-services-hand-upper-extremity/post-trauma	Private Service	USA	Medication; HT; CT;	No	No	No	No	0	Not Found	Not Found	0	
https://sportdoctorlondon.com/wrist-pain-and-popping/	Private Service	England	Medication;	No	No	No	No	0	28/1/2021	Not Found	0	
https://twinboro.com/why-does-my-wrist-hurt	Private Service	USA	Exercise; MT; CT;	No	No	No	No	0	8/5/2020	Not Found	0	
https://yorkvillephysiotherapy.com/why-does-the-pinky-side-of-my-wrist-hurt/	Private Service	Canada	Splinting;	No	Yes	No	No	5	Not Found	Not Found	2	
https://www.buoyhealth.com/learn/wrist-pain	Medical info outlet	USA	Medications; PT;	No	No	No	No	8	18/6/2022	Not Found	1	
https://www.wikihow.com/Relieve-Wrist-Pain-from-Lifting	Medical info outlet	USA	Medication; Taping;	Yes	No	No	No	0	4/12/2022	Not Found	1	
